# The human nucleoporin Tpr protects cells from RNA-mediated replication stress

**DOI:** 10.1038/s41467-021-24224-3

**Published:** 2021-06-24

**Authors:** Martin Kosar, Michele Giannattasio, Daniele Piccini, Apolinar Maya-Mendoza, Francisco García-Benítez, Jirina Bartkova, Sonia I. Barroso, Hélène Gaillard, Emanuele Martini, Umberto Restuccia, Miguel Angel Ramirez-Otero, Massimiliano Garre, Eleonora Verga, Miguel Andújar-Sánchez, Scott Maynard, Zdenek Hodny, Vincenzo Costanzo, Amit Kumar, Angela Bachi, Andrés Aguilera, Jiri Bartek, Marco Foiani

**Affiliations:** 1 IFOM, Fondazione Istituto FIRC di Oncologia Molecolare, Milano, Italy; 2 Institute of Molecular Genetics of the Czech Academy of Sciences, Prague, Czech Republic; 3 Universita’ degli Studi di Milano, Milano, Italy; 4 Danish Cancer Society Research Center, Copenhagen, Denmark; 5 Centro Andaluz de Biología Molecular y Medicina Regenerativa—CABIMER, Universidad de Sevilla-CSIC, Seville, Spain; 6 Division of Genome Biology, Department of Medical Biochemistry and Biophysics, Science for Life Laboratory, Karolinska Institute, Stockholm, Sweden; 7 Pathology Department, Complejo Hospitalario Universitario Insular Materno Infantil, Las Palmas de Gran Canaria, Spain; 8 Genome & Cell Integrity Laboratory, CSIR-Indian Institute of Toxicology Research, Lucknow, India; 9 Present Address: Division of Genome Biology, Department of Medical Biochemistry and Biophysics, Science for Life Laboratory, Karolinska Institute, Stockholm, Sweden

**Keywords:** Cancer, DNA replication, RNA metabolism

## Abstract

Although human nucleoporin Tpr is frequently deregulated in cancer, its roles are poorly understood. Here we show that Tpr depletion generates transcription-dependent replication stress, DNA breaks, and genomic instability. DNA fiber assays and electron microscopy visualization of replication intermediates show that Tpr deficient cells exhibit slow and asymmetric replication forks under replication stress. Tpr deficiency evokes enhanced levels of DNA-RNA hybrids. Additionally, complementary proteomic strategies identify a network of Tpr-interacting proteins mediating RNA processing, such as MATR3 and SUGP2, and functional experiments confirm that their depletion trigger cellular phenotypes shared with Tpr deficiency. Mechanistic studies reveal the interplay of Tpr with GANP, a component of the TREX-2 complex. The Tpr-GANP interaction is supported by their shared protein level alterations in a cohort of ovarian carcinomas. Our results reveal links between nucleoporins, DNA transcription and replication, and the existence of a network physically connecting replication forks with transcription, splicing, and mRNA export machinery.

## Abstract (web summary)

Tpr nucleoporin is known to be essential for nuclear transport and mitosis processes. Here the authors explore the link between Tpr and genome instability providing insights into the role of Tpr in safeguarding cells from RNA-mediated replication stress.

## Introduction

Failure to adequately respond to genotoxic stress may have catastrophic consequences at the cellular and even organismal level, leading to genomic instability and grave pathologies such as developmental defects, neurodegeneration, immunodeficiency, premature aging, or cancer^1^. Whereas inherited mutations in DNA repair genes may predispose to cancer^2^, endogenous replication stress, acquired DNA damage checkpoint defects, and other DNA damage response (DDR) abnormalities are common among sporadic tumors, fueling chromosomal instability, cancer cell heterogeneity, and thereby resistance to treatment and tumor progression^3,4^. At the same time, such tumor-specific DDR defects may unmask vulnerabilities exploitable in targeted cancer therapies^5^.

Among the emerging aspects of the DDR concept is the realization that genome integrity maintenance involves an intimate interplay with RNA metabolism, as well as proper functional compartmentalization and regulated transport between the nucleus and cytoplasm. Trafficking between the cytoplasm and nucleoplasm occurs through nuclear pores spanning the nuclear envelope, formed by nuclear pore complexes (NPC), cylindrical assemblies of evolutionarily highly conserved proteins known as nucleoporins^6^. Apart from their canonical role in nuclear transport, nucleoporins have been implicated in additional fundamental cellular processes such as gene regulation through chromatin silencing, transcriptional control, and RNA transactions^7^. Furthermore, abnormalities of diverse human nucleoporins have been associated with developmental defects, autoimmune dysfunction, neurological and cardiovascular disorders, and a range of malignancies^8^. The latter scenario reflects chromosomal translocations generating fusion proteins, point mutations, and altered protein levels and aberrations that lead to mislocalization or malfunction of such affected nucleoporins^8,9^. Notably, so far only a handful of nucleoporins, mainly those that share involvement in mRNA export, have been implicated in cancer, including Nup88, Nup98, Nup214, and translocated promoter region (Tpr)^10^. Based on our current knowledge, Tpr is implicated in cancer through several types of abnormalities, including: (i) chromosomal translocations generating fusion proteins with various receptor tyrosine kinases^11^; (ii) aberrant changes in mRNA levels^9^; and (iii) point mutations found in various types of cancer, although their functional significance has not been examined so far^9^.

Human nucleoporin Tpr is a large protein of ~267 kDa that contains a coiled-coil N-terminal segment capable of forming homodimers, and a highly posttranslationally modified C terminus^12^. The N-terminal domain tethers Tpr via interaction with Nup153 to the NPC, forming a basket-like structure on the nucleoplasmic side of the pore^13^. Beside Tpr main localization at NPCs, a nucleoplasmic fraction of Tpr was also detected in human cells^12,14^. Similarly, Tpr homologs in *Saccharomyces cerevisiae* (Mlp1 and Mlp2) and *Drosophila melanogaster* (Megator) localize to both NPCs and throughout the nuclear interior^15,16^, and the yeast Mlp proteins participate in RNA export and processing^17^ in a DNA replication checkpoint pathway and contribute to maintain genome stability by preventing harmful R-loop accumulation^18,19^. Human Tpr is multifunctional, contributing to NPC-mediated nuclear transport, yet also providing a mitotic scaffold in regulation of the spindle assembly checkpoint (SAC)^20^ and modulating chromatin organization, the latter role also impacting viral infections associated with altered chromatin states^21^. In terms of nucleocytoplasmic transport, some studies concluded that Tpr was required for nuclear protein export^14^, while others reported that both nuclear protein import and export remained functional in Tpr-depleted cells^22^. Recently, it has been shown that Tpr is required for the efficient nuclear export of intronless and intron-poor mRNAs and lncRNAs^23^ and that the acute depletion of Tpr using auxin-induced degron system causes changes in transcriptomic profile^24^. Collectively, these data suggest some functional relationship(s) between Tpr and RNA processing in mammalian cells, however the precise function of Tpr remains unknown.

Related to genome integrity, Tpr has been identified in a genome-wide siRNA screen among factors whose downregulation evoked elevated phosphorylation of the histone variant H2AX (γH2AX), an early mark of DNA damage^25^. Moreover, both human Tpr and its mouse homolog have been previously identified in a large-scale proteomic analysis of proteins phosphorylated in response to DNA damage on SQ/TQ consensus motifs recognized by the key upstream DDR kinases ATM and ATR^26^. Subsequent studies also found those and additional phosphorylation sites in Tpr to be regulated by DNA damage in different human cell types exposed to DNA-damaging insults including ultraviolet light (UV)^27^, ionizing radiation (IR)^28^, and a topoisomerase II inhibitor etoposide^29^. In a phospho-proteomic screen combined with chemical genetics, Tpr was identified as a substrate of Chk1^30^, a major DDR effector kinase activated by ATR^1^. Furthermore, additional screens have identified Tpr as a protein binding DNA, including association with nascent DNA molecules in human cells pulsed with EdU^31^, and in proteomic characterization of the human replisome and replisome-associated factors among proteins on nascent DNA (so-called iPOND-MS approach), with Tpr enriched in the proximity to the replication forks^32^.

It is currently unclear whether, and mechanistically how, Tpr might contribute to the maintenance of genomic integrity. Here, we propose that Tpr and its interacting partners (partly also identified here) act at the interface of transcription and DNA replication, contributing to genome stability in human cells exposed to replication stress, a common threat implicated in aging and an emerging hallmark of cancer.

## Results

### Tpr promotes cell survival and prevents DNA damage under replication stress

**Fig. 1:**
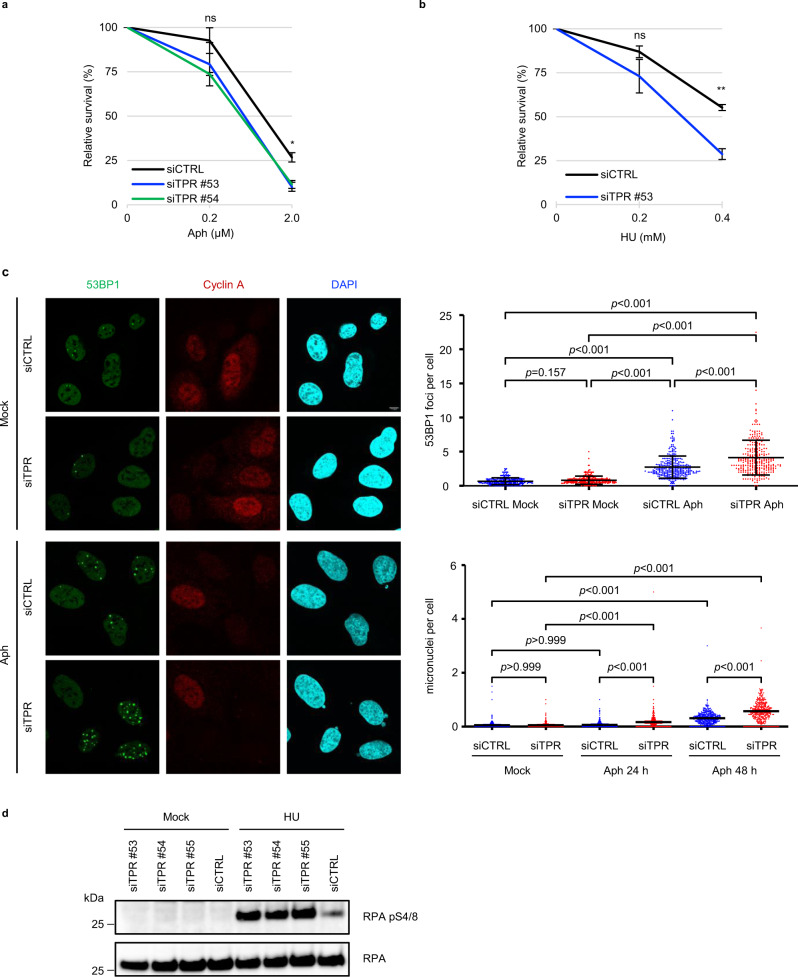
Tpr depletion sensitizes cells to replication stress. **a** Clonogenic survival of Tpr-depleted (siTPR) and control U-2 OS cells (siCTRL) treated with aphidicolin (Aph). Data are represented as mean ± SEM from three independent experiments (*n* = 3). Statistical analysis: Brown–Forsythe and Welch ANOVA with Dunnett’s T3 post analysis for multiple comparisons; ns not significant; *p value < 0.05. **b** Clonogenic survival of Tpr-depleted (siTPR) and control U-2 OS cells (siCTRL) treated with hydroxyurea (HU). Data are represented as mean ± SEM from three independent experiments (*n* = 3). Statistical analysis: Mann–Whitney test and Welch’s *t*-test; ns not significant; **p value < 0.01. **c** Representative images of 53BP1 foci in Tpr-depleted (siTPR) and control (siCTRL) U-2 OS cells. Cells were either nontreated (Mock) or treated with 0.2 μM aphidicolin for 48 h (Aph). The staining of cyclin A serves as a marker of the cell cycle stage. Nuclei were stained with DAPI. The scale bar, 7.5 µm. Top right: quantification of 53BP1 foci in Tpr-depleted (siTPR) and control U-2 OS cells (siCTRL) either nontreated (Mock) or exposed to 0.2 μM aphidicolin for 48 h (Aph). A series of nonoverlapping frames were randomly acquired by automated microscopy, and the 53BP1 foci in G1 nuclei (cyclin A negative) were counted. Data are represented as mean ± SD from three independent experiments (*n* = 3). Statistical analysis: Kruskal–Wallis test with Dunnett’s T3 post analysis for multiple comparisons. Bottom right: quantification of micronuclei in Tpr-depleted (siTPR) and control U-2 OS cells (siCTRL) performed on the same samples as 53BP1 foci analysis (top right panel). Data are represented as mean ± SEM from three independent experiments (*n* = 3). Statistical analysis: Kruskal–Wallis test with Dunnett’s T3 post analysis for multiple comparisons. **d** Depletion of Tpr leads to increased RPA pS4/8 phosphorylation upon hydroxyurea treatment. Cells were transfected with three independent siRNAs targeting Tpr (siTPR #53, siTPR #54, siTPR #55) or control siRNA (siCTRL). Transfected cells were either nontreated (Mock) or treated with 2 mM hydroxyurea (HU) for 4 h. Staining of RPA total is used as a loading control. Data are representative of three independent experiments. Source data for **d** are provided in the Source Data file.

To investigate the potential role of Tpr in the maintenance of genome stability, we first assessed responses of Tpr-depleted human U-2 OS cells to drugs inducing replication stress. The robust Tpr knockdown was confirmed using immunoblotting (Supplementary Fig. 1a). Compared to Tpr-proficient control cells, siRNA-mediated Tpr knockdown resulted in reduced clonogenic cell survival under exposure to DNA replication inhibitors aphidicolin (Aph) and hydroxyurea (HU) (Fig. 1a, b). The observed hypersensitivity phenotype of Tpr-depleted cells was rescued by expression of a siRNA-insensitive wild-type Tpr, thereby excluding any off-target effect of our siRNAs (Supplementary Fig. 1b). These results indicated that Tpr promotes cell survival and growth under replication stress.

Next, we exposed the Tpr-depleted and control cells to low doses of Aph (0.2 μM) for 48 h, a scenario previously shown to selectively impair progression of DNA replication forks through physiological barriers such as common fragile sites^33^. As a phenotypic read-out, we examined the frequency of G1-phase nuclear bodies marked by a double-stranded DNA break (DSB) response protein 53BP1, an assay widely used to document the impact of replication stress experienced by cells in their previous cell cycle^34^. While in Tpr-proficient cells Aph treatment resulted in the expected increase of 53BP1 bodies in G1 (cyclin A negative) cells compared to untreated controls, a parallel examination of Tpr-depleted Aph-treated cells showed a further increased number of G1 53BP1 bodies and also micronuclei, a well-established marker of genome instability (Fig. 1c). Thus, Tpr deficiency enhances the extent and consequences of cellular replication stress.

The clonogenic survival assays indicated that loss of Tpr sensitizes cells to mild inhibition of DNA replication, thereby suggesting a function for Tpr that is distinct from its known role in mitosis as a regulator of the SAC^20^. To distinguish the mitotic role of Tpr from its possible novel function in *S* phase, we exposed human cells to a short 4-h HU treatment, and then assessed phosphorylation of the replication protein A at serine residues 4 and 8 (pRPA S4/8), a replication stress-associated event that signals the presence of extended single-stranded DNA (ssDNA)^35^. Such 4-h exposure to 2 mM HU-evoked robust pRPA S4/8 in U-2 OS cells depleted of Tpr by 3 different siRNAs, respectively, in contrast to only a modest pRPA S4/8 signaling in control Tpr-proficient cells (Fig. 1d). Simultaneously, we did not observe any differences in Chk1 and ATR protein levels or their phosphorylation between Tpr-depleted and control cells (Supplementary Fig. 1c, d).

Taken together, these results suggested that Tpr deficiency may impair progression of DNA replication forks, especially under replication stress, a notion that we set out to test next.

### Tpr deficiency generates asymmetric forks and aberrant fork progression under replication stress

**Fig. 2:**
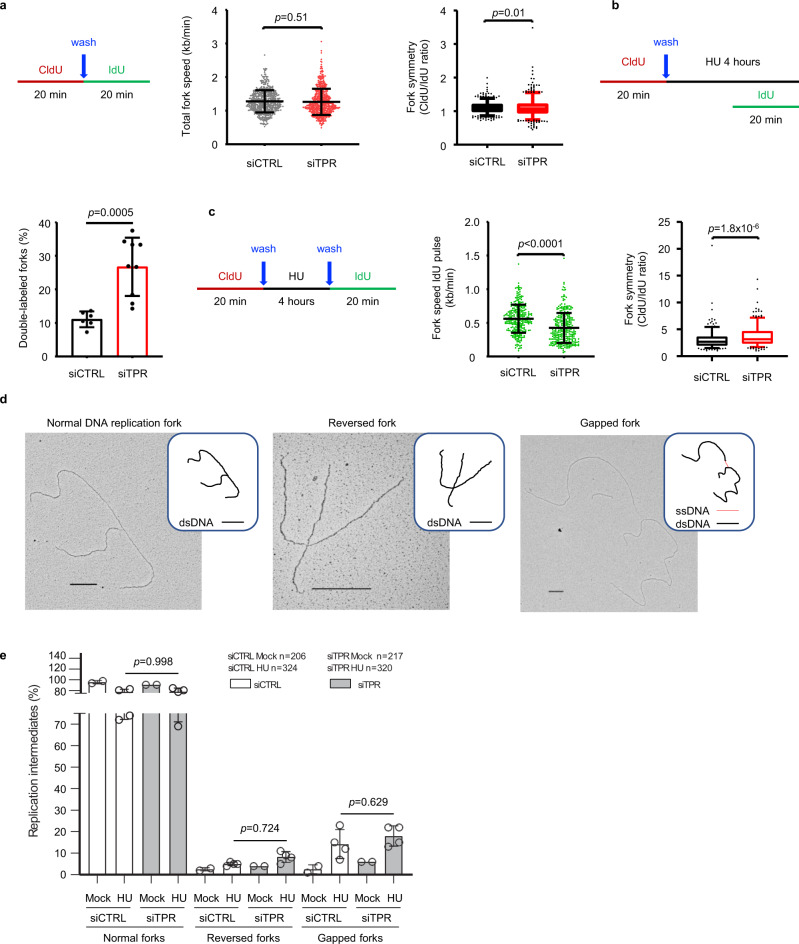
Tpr depletion impacts replication fork progression. **a** DNA fiber analysis of the replication fork speed and symmetry in nontreated conditions. Left: schematic of the CldU/IdU pulse-labeling protocol used. Middle: analysis of the replication fork speed. Total number of molecules analyzed: siCTRL (*n* = 541), siTPR (*n* = 567). The plot includes the mean ± SD. Statistical test: unpaired *t*-test with Welch’s correction, two-tailed. Right: analysis of the replication fork symmetry. The ratio between CldU/IdU was calculated from values in **a** (left). Whiskers indicate the 5th–95th percentiles (*p* value associated with Kolmogorov–Smirnov test). **b** DNA fiber analysis of the ability of forks to stop progression in the presence of replication stress. Top: schematic of the CldU/IdU pulse-labeling protocol used. Bottom: percentage of the double-labeled replication forks containing both CldU and IdU signal is showed. Total number of slides analyzed from three independent experiments: siCTRL (*n* = 6), siTPR (*n* = 9). The plot includes the mean ± SD. Statistical test: unpaired *t*-test with Welch’s correction, two-tailed. **c** DNA fiber analysis of the replication fork recovery upon HU removal. Left: schematic of the CldU/IdU pulse-labeling protocol used. Middle: length of the IdU tracks measured upon hydroxyurea removal. Total number of molecules analyzed: siCTRL (*n* = 297), siTPR (*n* = 310). The plot includes the mean ± SD. Statistical test: unpaired *t*-test with Welch’s correction, two-tailed. Right: analysis of the replication fork symmetry. The ratio between CldU/IdU was calculated from values in **c** (middle). Whiskers indicate the 5th–95th percentiles. Statistical test: unpaired *t*-test with Welch’s correction, two-tailed. **d** Categories of DNA replication intermediates identified in TEM analysis with a schematic representation of the molecule with dsDNA in black and ssDNA in red. Scale bars of 360 nm, which correspond to 1 kb of DNA, are reported on each picture. **e** Chart reporting the percentages of RIs identified. Gapped forks are defined as forks with ssDNA gaps at fork junction points longer than 200 nts. Data are represented as mean ± SD from four independent experiments (*n* = 4) for hydroxyurea-treated (HU) samples and two independent experiments (*n* = 2) for nontreated samples. Statistical analysis: two-way ordinary ANOVA followed by Sidak’s multiple comparison test.

To gain a more direct insight into the emerging function of Tpr in DNA replication, we used the DNA fiber analysis approach, which allows assessment of replication fork dynamics at the single-molecule resolution level^36^. First, we compared the speed of replication fork progression in human Tpr-depleted and control cells under unperturbed growth conditions. U-2 OS cells were pulse-labeled with 5-chlorodeoxyuridine (CldU; red) for 20 min, washed and pulse-labeled with 5-iododeoxyuridine (IdU; green) for another 20 min, followed by DNA spreading and standard immunofluorescence analysis. After measuring the length of labeled tracks (CldU and IdU), fork speed was calculated by dividing the track length by the labeling time. Under such conditions, we did not observe any significant differences in the replication fork speed between Tpr-depleted and control cells (Fig. 2a). However, a modest yet statistically significant increase in the frequency of asymmetric forks became apparent in the Tpr-depleted cells, as evaluated by calculating the CldU/IdU ratios (Fig. 2a). In Tpr-depleted HeLa cells, we observed an even larger effect on asymmetry (Supplementary Fig. 2). Since fork asymmetry represents a measure of replication fork arrest, these results suggested a possible role of Tpr in preventing fork stalling/collapse.

Next, we tested the ability of the replication forks to pause progression under HU-evoked inhibition of DNA synthesis that reflects imbalanced deoxyribonucleotide pools^37^. Tpr-depleted and control cells were pulse-labeled with CldU (red) for 20 min, washed, and treated with 2 mM HU for 4 h. IdU (green) was added directly into the HU-containing medium for the last 20 min of the HU treatment. Given the global replication-stalling effect of HU^36^, most forks are expected to stall their progression, and those unable to stop become double-labeled since both CldU (red) and IdU (green) are incorporated. These experiments showed a higher percentage of double-labeled forks in the Tpr-depleted cells than in control cells (Fig. 2b), indicating an aberrant inability of replication forks in Tpr-depleted cells to properly arrest their progression under continuous presence of HU.

Next, we evaluated the ability of transiently stalled forks to restart their progression. Tpr-depleted and control cells were pulse-labeled with CldU (red), washed, exposed to 2 mM HU for 4 h, and after switch to HU-free medium, the cells were pulse-labeled with IdU (green) for 20 min. Replication fork restart was evaluated by measuring the fork speed deduced from the length of IdU (green) tracks, which turned out to be shorter in Tpr-depleted cells (Fig. 2c). We concluded that Tpr depletion undermines the timely restart of replication forks transiently arrested under replication stress. Notably, parallel assessment of replication fork asymmetry by calculating CldU/IdU ratios in the latter experiments revealed a pronounced degree of fork asymmetry in Tpr-depleted cells compared with Tpr-proficient counterpart cells (Fig. 2c), an abnormality that was strongly accentuated by pretreatment with HU.

Overall, our results obtained with the DNA fiber analyses revealed that Tpr deficiency results in a moderately increased fork asymmetry under normal growth conditions and highly abnormal function (inability to timely stop and restart, and high degree of asymmetry) of replication forks under replication stress. In principle, fork asymmetry can result from extensive fork stalling or even fork collapse and the same defects can account for the inability of Tpr-depleted forks to recover from the HU block. However, the observation that Tpr-defective cells exhibit unscheduled DNA synthesis following a prolonged HU-induced replication block is apparently at odds with a high incidence of extensive fork stalling/collapse. An alternative interpretation is that Tpr-depleted cells experiencing replication stress exhibit aberrant restart events downstream of HU-blocked forks.

To further characterize the replication defects of TPR-depleted cells, we combined in vivo psoralen cross-linking and transmission electron microscopy (EM), a well-established methodology to analyze replication intermediates (RIs) in mammalian cells^38^. Using this technique, it is possible to identify different classes of RIs at the fork level, such as normal forks, characterized by three-branched molecules, reversed forks, resulting from fork collapse and gapped forks, exhibiting either leading-lagging uncoupling and/or selective resection of nascent chains^39^ (Fig. 2d). In untreated Tpr-depleted and control U-2 OS cells, we observed normal forks, reversed forks, and gapped forks with comparable frequencies. Next, we exposed control and Tpr-depleted U-2 OS cells to 2 mM HU for 4 h. The HU-evoked replication stress enhanced the frequency of gapped and reversed forks at comparable rates in Tpr-depleted and control cells (Fig. 2e). Hence, Tpr-deficient cells did not exhibit increased fork collapse or aberrant fork intermediates compared to control cells, not even when cells experienced replication stress.

### R-loop accumulation and transcription-dependent DNA damage upon Tpr depletion

**Fig. 3:**
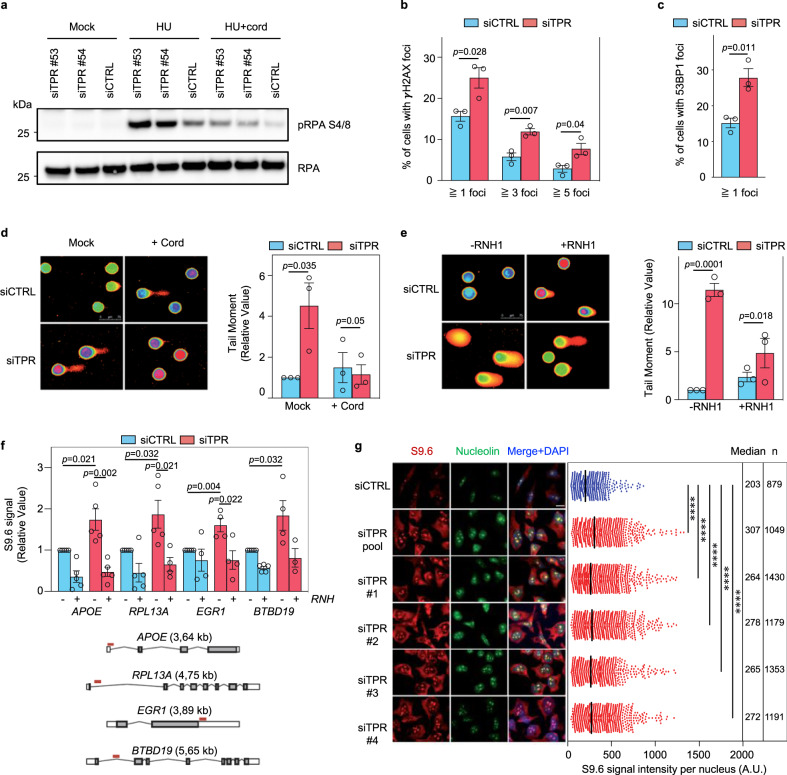
Tpr depletion leads to DNA–RNA hybrid accumulation. **a** Tpr-depleted (siTPR) and control (siCTRL) cells. Treatments: nontreated (Mock); 2 mM hydroxyurea 4 h (HU); 50 μM cordycepin 3 h followed by 2 mM HU 4 h (HU + cord). RPA total, loading control. Data are representative of three independent experiments. Source data are provided in the Source Data file. **b** Quantification of γH2AX-foci in siTPR (red) and siCTRL (blue) HeLa cells. Data represent mean ± SEM from independent experiments (*n* = 3). Statistical analysis: two-tailed unpaired Student’ *t* test. **c** Quantification of 53BP1 foci in siTPR and siCTRL HeLa cells. Other details as in **b**. **d** DNA breaks in Tpr-depleted cells are transcription dependent. Alkaline comet assay in mock or cordycepin-treated (+Cord) siTPR (red) or siCTRL (blue) HeLa cells. Representative images (left) and mean ± SEM of median comet tail moment (right) from independent experiments (*n* = 3). Statistical analysis: two-tailed unpaired Student’ *t* test. **e** RNaseH1 expression rescues DNA breaks in Tpr-depleted HeLa cells. Alkaline comet assay in siTPR (red) and siCTRL (blue) cells expressing RNaseH1 from plasmid pEGFP-M27-H1 (+RNH1) or transfected with plasmid pEGFP-N2 (−RNH1). Other details as in **d**. **f** DNA–RNA hybrid immunoprecipitation with the S9.6 antibody in siTPR (red) and siCTRL (blue) HeLa cells at the *APOE*, *RPL13A*, *EGR1*, and *BTBD19* genes. Where indicated samples were treated with RNase H (RNH) prior to immunoprecipitation. S9.6 signal values were normalized to the control cells value of each locus in each experiment. Location of the amplicon relative to each gene is indicated. Mean ± SEM of signal values of R-loop detection obtained from independent experiments are shown (*n* = 5, except for siTPR +RNH of *RPL13A*, siCTRL +RNH, and both siTPR of *EGR1*, siTPR −RNH of BTBD19 for which *n* = 4, and siTPR +RNH of BTBD19 for which *n* = 3). Statistical analyses with a two-tailed unpaired Student’ *t* test. **g** siTPR pool deconvolution analysis by S9.6 immunofluorescence in HeLa cells. Immunofluorescence with S9.6 and anti-nucleolin antibodies in siCTRL (blue), siTPR pool (siTPR pool; red), or each individual siRNAs of the siTPR pool (siTPR #1, siTPR #2, siTPR #3, siTPR #4; red) transfected cells. Representative images from three independent experiments (left) and quantification of S9.6 signal intensity per nucleus after the subtraction of the nucleolar signal (right). Scale bar, 7.5 µm. Median value and number of cells (*n*) examined over three independent experiments are indicated. Statistical analysis: two-tailed unpaired Mann–Whitney *U* test; ****p < 0.0001.

Given that a common cause of replication stress is *S* phase transcription, we tested whether the observed phenotypes under Tpr deficiency are related to transcription in human cells, using the regularly used transcription inhibitors cordycepin and DRB^40,41^. As expected, in the samples treated only with HU, Tpr depletion led to a higher level of RPA S4/8 phosphorylation than in control cells. This phenotype was rescued both in Tpr-depleted and control U-2 OS cells by pretreatment with cordycepin and DRB before exposure of cells to HU (Fig. 3a; Supplementary Fig. 3a).

To extend and validate our observations in another human cell type, we then used Tpr-depleted or control Tpr-proficient HeLa cells, a model known for its enhanced endogenous replication stress. As documented by the immunofluorescence analysis of the foci formed by the established DNA damage markers γH2AX and 53BP1, endogenous DNA damage was enhanced in Tpr-depleted HeLa cells, compared to their control counterparts (Fig. 3b, c; Supplementary Fig. 3b). Next, we employed single-cell electrophoresis (comet assay) performed under alkaline and neutral conditions to detect both single-stranded breaks and DSBs or only DSBs, respectively. With both techniques, we observed that Tpr depletion in HeLa cells, without any additional treatment, resulted in a higher incidence of DNA breaks than in control cells (Supplementary Fig. 3c). To investigate whether the accumulation of DNA damage in Tpr-depleted HeLa cells is also transcription dependent, as it is in U-2 OS cells, we again used the transcription inhibitor cordycepin. The extent of DNA breaks in Tpr-depleted cells was reduced in the samples treated with cordycepin, suggesting that the origin of a sizeable subset of DNA breaks required transcription (Fig. 3d).

A plausible contributor to the observed DNA breakage in Tpr-depleted cells was an increased accumulation of DNA–RNA hybrids, which can generate R-loops and genotoxic events^42^. To test this possibility, we transfected Tpr-depleted and control HeLa cells with a vector expressing RNaseH1, an enzyme capable of digesting the RNA moiety of DNA–RNA hybrids^40^. Measurement of the tail moment by alkaline comet assay revealed that expression of RNaseH1 could rescue the phenotype of enhanced DNA breaks in Tpr-depleted HeLa cells (Fig. 3e). This suggests that DNA–RNA hybrids are responsible for the breaks. Consequently, we determined the levels of R-loops in Tpr-depleted and control cells using DNA–RNA hybrid immunoprecipitation (DRIP) with the S9.6 antibody at the transcribed genes *APOE*, *RPL13A*, *EGR1*, and *BTBD19*. In all four genes tested, the Tpr-depleted cells accumulated R-loops at a statistically significant higher level than control cells. In samples treated with RNase H, the levels of DNA–RNA hybrids decreased, confirming that the signal detected was specific for DNA–RNA hybrids (Fig. 3f).

In addition to the DRIP approach, we also confirmed the preferential accumulation of DNA–RNA hybrids in Tpr-depleted cells by immunofluorescence, including the siTPR pool deconvolution analysis by S9.6 immunofluorescence (Fig. 3g). It is worth noticing that in our experience, HeLa cells are more sensitive for R-loop detection than U-2 OS cells. This difference could be related to the RB and p53 inactivation in HeLa, the ALT mechanism of telomere elongation operating only in U-2 OS cells or other conditions that would make HeLa cells distinct and more sensitive to depletion of specific factors such as Tpr. R-loops could go higher in HeLa due to a different background level of replication stress (likely reflecting the HPV E7-mediated RB inactivation and hence deregulated *S* phase entry), as supported by a higher level of 53BP1 in HeLa cells (Fig. 3c compared to Fig. 1c) and replication fork asymmetry (Supplementary Fig. 2 compared to Fig. 2a).

### Identification and functional annotation of Tpr-interacting proteins

To identify the molecular pathways in which Tpr operates and to provide mechanistic insights into the phenotypes that we observed so far, we next searched for proteins interacting with Tpr in human cells, by combining stable isotope labeling with amino acids in cell culture (SILAC)^43^, with high-throughput identification of proteins by mass spectrometry and statistical analysis. We used three different experimental setups to maximize the accuracy of our results.

In the first setup, we analyzed Tpr protein interactions in U-2 OS cells transiently transfected either with the vector expressing Tpr-GFP or with an empty vector as a negative control, so that Tpr-GFP or GFP were immunoprecipitated. In the second setup, we compared U-2 OS cells expressing endogenous Tpr (cells transfected with siRNA control) versus U-2 OS cells depleted of endogenous Tpr as a negative control (cells transfected with siRNA TPR). Cell extracts from both cultures were subjected to Tpr immunoprecipitation using the antibody recognizing endogenous Tpr. In the third experimental scenario, while cells in both samples expressed only endogenous Tpr, the cell extract from the first sample was subjected to immunoprecipitation using the antibody recognizing endogenous Tpr, and the extract from the second sample was immunoprecipitated using an IgG antibody as a negative control. A total of 1921, 671, and 831 proteins were identified in the three setups, respectively (Supplementary Table 1).

Overall, using stringent selection criteria (see “Methods” section), we selected proteins that were identified in at least two out of our three SILAC approaches. To the list of those nine selected proteins, we added GANP protein, which was recognized in one of our SILAC experiments, and also in our previously conducted label-free mass-spectrometry analysis without the introduction of stable isotopes performed in the same cell type. The list of selected Tpr-interacting proteins is provided in Table 1.

**Table 1:** Tpr interacts with proteins involved in RNA metabolism.

Protein		Median ratios		
		SILAC I	SILAC II	SILAC III
Matrin 3	MATR3	145.73	14.89	109.12
SURP and G-patch domain containing 2	SUGP2	32.93	10.59	50.01
Heterogeneous nuclear ribonucleoprotein C	HNRNPC	17.68	3.38	6.08
RALY heterogeneous nuclear ribonucleoprotein	RALY	14.87	3.35	5.82
Heterogeneous nuclear ribonucleoprotein M	HNRNPM	11.93	3.43	9.27
Zinc finger protein 326	ZNF326	17.24	x	5.86
Translocated promoter region	TPR	7.06	x	61.98
Nucleoporin 153	NUP153	6.81	x	2.97
ELAV like RNA binding protein 1	ELAVL1	5.88	x	5.44
Polypyrimidine tract binding protein 1	PTBP1	x	5.33	8.11
Minichromosome maintenance complex component 3 associated protein	GANP (MCM3AP)	x	4.56	x

The list of selected Tpr-interacting proteins identified in the SILAC experiments. The median ratios of the significant proteins identified as Tpr interactors are presented. “x” stands for protein not identified.

To decipher the biological and functional context of the most significant proteins identified to be interacting with Tpr in our experiments (Table 1), we performed protein network and functional enrichment analysis using the STRING database^44^. Our protein network analysis revealed that Tpr protein interactors have more connections among themselves than what would be expected for a random set of proteins of similar size, suggesting that the proteins are at least partially biologically connected. Notably, Tpr-interacting protein network is enriched in proteins involved in RNA metabolism. At least seven proteins from our network (SUGP2, heterogenous nuclear ribonucleoprotein C (HNRNPC), RALY, HNRNPM, ZNF326, PTBP1, GANP) are known factors implicated in RNA processing, eight proteins can bind poly(A)+RNA (MATR3, SUGP2, HNRNPC, RALY, HNRNPM, ZNF326, ELAVL1, PTBP1) and four proteins (HNRNPC, HNRNPM, ELAVL1, PTBP1) harbor RNA recognition motifs (RRM, RBD, or RNP domains).

Furthermore, several proteins in our network were previously linked to genome integrity maintenance. Thus, the HNRNPC is a core component of 40S ribonucleoprotein particles that coat nascent pre-mRNAs and impact their processing, stability, and export^45^. In addition, HNRNPC participates in the regulation of alternative splicing^46^ and contributes to DNA repair through interaction with the known repair proteins PALB2, BRCA1, and BRCA2. Following IR, a fraction of HNRNPC becomes recruited to DNA damage sites, and depletion of HNRNPC impairs DSB repair^45^. MATR3 and HNRNPM are splicing factors and substrates of the DDR checkpoint kinase Chk1^30^. MATR3 interacts with PTBP1^47^ and regulates retention of SFPQ/NONO at DNA damage sites^48^.

To further validate the results of the high-throughput proteomic screens, we performed a standard coimmunoprecipitation assay. We confirmed the physical interaction between Tpr and MATR3 (Supplementary Fig. 4a), and Tpr and GANP (Supplementary Fig. 4b).

### Replication stress-sensitive phenotypes are shared upon depletion of Tpr or its interactors

**Fig. 4:**
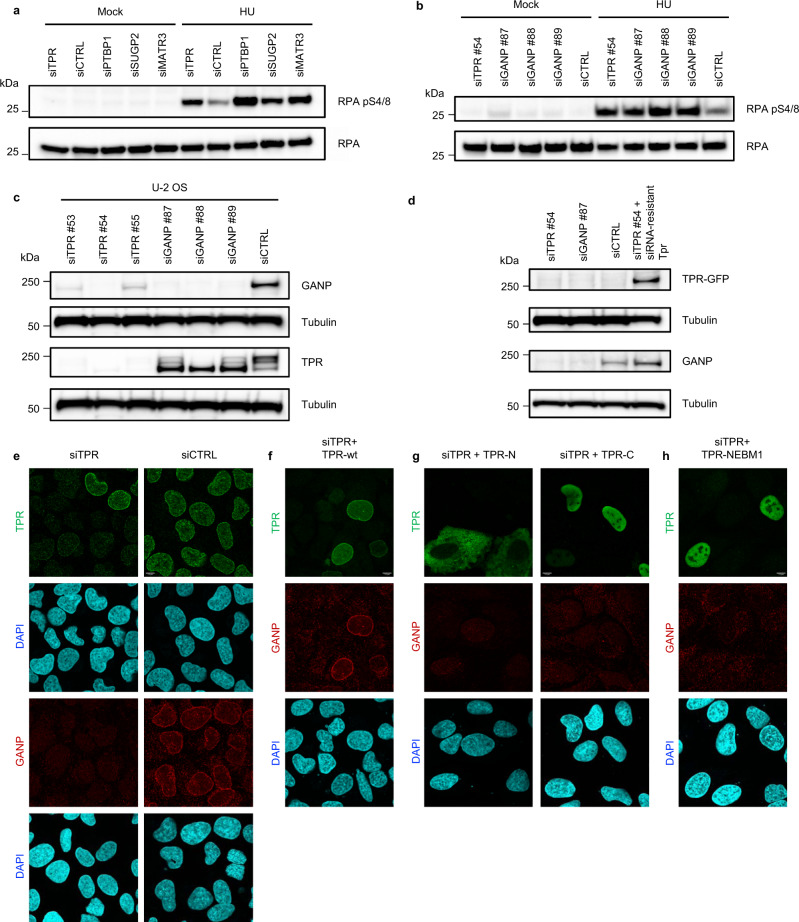
Tpr maintains normal protein levels of GANP. **a** U-2 OS cells were transfected using siRNAs and either nontreated (Mock) or treated with 2 mM hydroxyurea (HU) for 4 h. RPA, loading control. Data are representative of three independent experiments. **b** U-2 OS cells were transfected using siRNAs and treated as in **a**. RPA, loading control. The experiment was performed twice yielding similar results. **c** U-2 OS cells were transfected using siRNAs. Tubulin, loading control. Data are representative of three independent experiments. **d** U-2 OS cells were transfected using siRNA targeting endogenous Tpr (siTPR #54), GANP (siGANP #87), or control siRNA (siCTRL). In parallel, cells were cotransfected with siRNA-resistant Tpr-GFP insensitive to siTPR #54. Tpr-GFP and GANP protein levels were analyzed by immunoblotting. Tubulin, loading control. The experiment was performed twice yielding similar results. **e** U-2 OS cells were transfected with siRNAs and levels of Tpr and GANP were assessed using immunofluorescence. Nuclei were stained with DAPI. The scale bar represents 7.5 µm. Data are representative of three independent experiments. **f** Expression of siRNA-resistant full-length Tpr-wt can rescue GANP protein level. U-2 OS cells were transfected with siRNA targeting endogenous Tpr (siTPR), and with siRNA-resistant Tpr-wt-GFP (TPR-wt). Levels of Tpr-wt-GFP and GANP were assessed using immunofluorescence. Nuclei, DAPI. The scale bar, 7.5 µm. Data are representative of three independent experiments. **g** Expressions of Tpr-N-terminal domain or Tpr-C-terminal domain are not able to rescue GANP protein level. U-2 OS cells were transfected with siRNA targeting endogenous Tpr (siTPR), and with Tpr-N-terminal-FLAG (TPR-N) or Tpr-C-terminal-FLAG (TPR-C) constructs. Levels of Tpr-FLAG and GANP were assessed using immunofluorescence. Nuclei, DAPI. The scale bar, 7.5 µm. Data are representative of three independent experiments. **h** Expression of siRNA-resistant full-length Tpr-NEBM (Nuclear Envelope Binding Mutant) is not able to rescue GANP protein level. U-2 OS cells were transfected with siRNA targeting endogenous Tpr (siTPR), and with siRNA-resistant Tpr-NEBM1-GFP (NEBM1) construct carrying L458P/M489P double amino acid substitution in Tpr nuclear envelope binding domain. Levels of Tpr-NEBM1-GFP and GANP were assessed using immunofluorescence. Nuclei, DAPI. The scale bar, 7.5 µm. Data are representative of three independent experiments. Source data for **a**–**d** are provided in the Source Data file.

Next, we tested whether depletion of proteins identified in our SILAC experiments as Tpr interactors may evoke replication stress sensitivity phenotypes reminiscent of those seen in Tpr-depleted cells. Indeed, cells knocked down for PTBP1, SUGP2, MATR3, and Tpr, respectively, all showed hyper-reactive phosphorylation of RPA pS4/8 under treatment with 2 mM HU for 4 h (Fig. 4a). At this point, we made two observations: (i) Tpr interacts with proteins involved in RNA processing and (ii) depletion of these proteins leads to the same phenotype as Tpr depletion in cells exposed to HU. Therefore, we concluded that Tpr might act in an RNA processing pathway and disruption of this pathway by Tpr depletion sensitizes human cells to replication stress.

Next, we focused on providing insights into the functional interplay of Tpr with GANP, for two reasons: (i) GANP is a component of the TREX-2 complex and thereby one of the Tpr interactors implicated in RNA processing^49^ and (ii) aberrations of *GANP* have been associated with grave diseases, including diverse forms of neuromuscular degeneration and cancer^50,51^, suggesting a potential relevance of the Tpr–GANP interaction in human pathophysiology. First, in the 4-h HU exposure assay analogous to the above experiments and phenotypes observed for Tpr and the other tested Tpr interactors (Fig. 4a), siRNA-mediated depletion of GANP also triggered a robust RPA S4/8 hyperphosphorylation response in U-2 OS cells (Fig. 4b). In addition, immunoblotting experiments showed that depletion of endogenous Tpr with any of the three different, individually applied, siRNAs led to a substantial decrease of endogenous GANP (Fig. 4c). This effect was not reciprocal since depletion of GANP by none of the individually applied siRNAs targeting endogenous GANP impacted the overall abundance of Tpr despite the GANP protein was reduced to almost undetectable levels in these experiments (Fig. 4c). The robust reduction of GANP levels upon depletion of its partner Tpr, established in the U-2 OS model (Fig. 4c) was then reproduced in three additional human cell types, including HeLa cells, benign breast epithelial cells (MCF10A), and normal diploid BJ fibroblasts in experiments that also confirmed the lack of any reciprocal impact on Tpr abundance upon depletion of GANP (Supplementary Fig. 5a). At the same time, the depletion of other identified Tpr interactors (MATR3, SUGP2, and PTBP1) did not impact GANP protein level (Supplementary Fig. 5b).

The specific nature of the destabilizing impact of Tpr depletion on GANP levels was then documented by the ability of ectopically expressed, full-length, wild-type, siRNA-resistant Tpr to rescue the level of endogenous GANP under conditions when the endogenous Tpr was knocked down by siRNA (Fig. 4d).

At the subcellular localization level, Tpr knock down resulted in a gross reduction of the GANP staining signal and a complete loss of GANP from its otherwise predominant localization at the nuclear envelope, while depletion of GANP altered neither the staining intensity nor the nuclear envelope localization of Tpr (Fig. 4e and Supplementary Fig. 5c), overall consistent with the immunoblotting results.

Structure-function analysis then addressed the issue of which part(s) of Tpr are essential for the support of GANP protein abundance, taking advantage of truncated or mutant versions of Tpr tagged by either FLAG or GFP for convenient tracking, the siRNA-resistant versions of which were ectopically expressed in U-2 OS cells depleted of endogenous Tpr, followed by combined immunofluorescence assessment of Tpr-FLAG/Tpr-GFP and GANP. These experiments established that in contrast to ectopic full-length wild-type Tpr, the expression of which rescued the protein level of endogenous GANP, ectopically expressed Tpr-N-terminal domain (amino acids 1–800), Tpr-C-terminal domain (amino acids 1626–2349), or either of the two full-length subtle mutants of Tpr that are unable to bind to the nuclear envelope (termed Tpr-NEBM1 and Tpr-NEBM2) were unable to rescue the protein levels of GANP (Fig. 4f–h and Supplementary Fig. 5d). Tpr nuclear envelope binding mutants (Tpr-NEBM1 and Tpr-NEBM2) having double amino acid substitutions (L458/M489 for proline or aspartic acid, respectively) within the nuclear envelope binding domain, through which Tpr protein is connected to the nuclear pore complex, were described previously^12^. Importantly, those full-length Tpr-NEBM mutants can enter into the nucleus, but they cannot bind to the nuclear envelope due to amino acid substitutions in the nuclear envelope binding domain. We conclude from these results that Tpr maintains normal protein levels of GANP, while GANP is dispensable for the abundance of Tpr. Interestingly, the decreased abundance of GANP protein induced by Tpr depletion is rescued by treatment with the proteasome inhibitor MG132 (Supplementary Fig. 5e). This suggests that loss of Tpr affects GANP levels through protein destabilization. Using RT-qPCR, we revealed that Tpr depletion does not impact the mRNA level of GANP (Supplementary Fig. 5f).

**Fig. 5:**
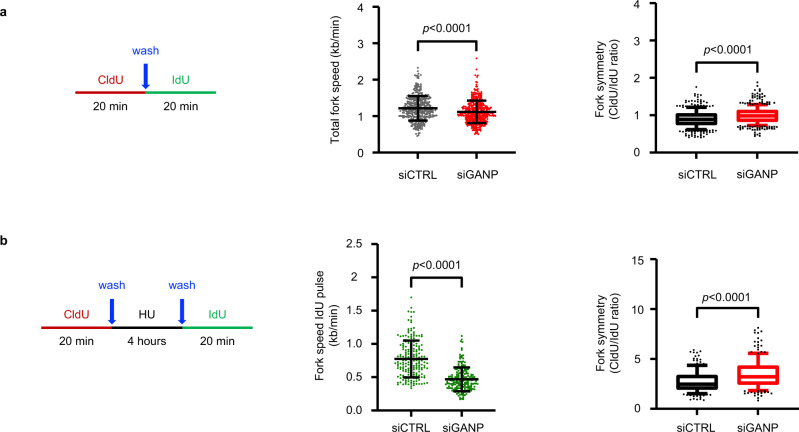
GANP-depleted cells exhibit replication defects. **a** DNA fiber analysis of the replication fork speed and symmetry in nontreated conditions. Left: schematic of the CldU/IdU pulse-labeling protocol used. Middle: analysis of the replication fork speed. Total number of molecules analyzed: siCTRL (*n* = 362), siGANP (*n* = 407). The plot includes the mean ± SD. Statistical test: unpaired *t*-test with Welch’s correction, two-tailed. Right: analysis of the replication fork symmetry. The ratio between CldU/IdU was calculated from the same molecules used for the analysis of the replication fork speed (middle) (siCTRL *n* = 362, siGANP *n* = 407). Bounds of box are 25th–75th percentile, center shows the median, whiskers indicate the 10th–90th percentiles, data outside the range are drawn as individual dots. Statistical analysis: unpaired *t*-test with Welch’s correction, two-tailed. **b** DNA fiber analysis of the replication fork recovery upon HU removal. Left: schematic of the CldU/IdU pulse-labeling protocol used. Middle: length of the IdU tracks measured upon hydroxyurea removal. Total number of molecules analyzed: siCTRL (*n* = 190), siGANP (*n* = 181). The plot includes the mean ± SD. Statistical test: unpaired *t*-test with Welch’s correction, two-tailed. Right: analysis of the replication fork symmetry. The ratio between CldU/IdU was calculated from the same molecules used for the analysis of the length of the IdU tracks measured upon hydroxyurea removal (middle) (siCTRL *n* = 190, siGANP *n* = 181). Bounds of box are 25th–75th percentile, center shows the median, whiskers indicate the 10th–90th percentiles, data outside the range are drawn as individual dots. Statistical analysis: unpaired *t*-test with Welch’s correction, two-tailed.

Next, we assayed replication fork progression and fork asymmetry in GANP-depleted cells using DNA fiber analysis as done for Tpr-depleted cells. We observed that in nontreated conditions, GANP depletion leads to slower replication fork speed and increased frequency of asymmetric forks (Fig. 5a). This result fits with the increased frequency of asymmetric forks of Tpr-depleted cells (Fig. 2a; Supplementary Fig. 2), even though in that case fork speed was unaffected. These results are consistent with previous observations that replication arrests cause fiber asymmetry, but do not necessarily result in slower replication fork speed as detected in long distances^52^. We then evaluated the ability of transiently stalled forks to restart their progression in GANP-depleted cells. The results revealed that GANP depletion undermines the timely restart of replication forks transiently arrested under replication stress conditions and that fork asymmetry in GANP-depleted is accentuated by pretreatment with HU (Fig. 5b), similar to Tpr-depleted cells (Fig. 2c).

DNA fibers experiments were complemented by analyses of the EdU profiles in Tpr- and GANP-depleted cells in both unchallenged and replication stress conditions as shown before (Figs. 2a–c and 5a, b). In accordance with the DNA fiber analysis, under untreated conditions, GANP depletion has a dramatic effect on the bulk of DNA synthesis, whereas Tpr-depleted cells exhibit a profile similar to normal cells (Supplementary Fig. 6). After HU treatment, the bulk of DNA synthesis was clearly affected in GANP-depleted cells, similarly to Tpr-depleted cells and normal cells, whereas GANP-depleted cells exhibit an inefficient restart of DNA synthesis following HU release and Tpr-depleted cells and normal cells show similar efficiency (Supplementary Fig. 6). We conclude that GANP-depleted cells, apart from an extensive fork pausing/stalling under normal conditions, shows a dramatic replication defect consistent with the known replication functions of GANP in modulating MCM3 and in priming DNA synthesis^53,54^. However, this phenotype was not observed in Tpr-depleted cells. Even though Tpr-depleted cells exhibit a partial reduction in GANP protein levels, it is insufficient to impact fork speed.

In summary, depletion of Tpr and any of its tested interacting proteins that are implicated in RNA processing, including GANP, evokes a shared phenotype of enhanced replication stress, a condition commonly seen in early as well as advanced stages of cancer.

### Relevance of Tpr and its interplay with GANP for human tumors and genomic (in)stability

**Fig. 6:**
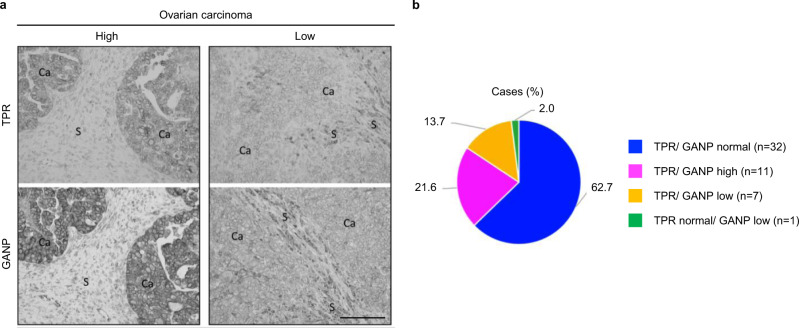
Analysis of Tpr and GANP protein levels in human tumors. **a** Immunohistochemical analysis of Tpr and GANP protein levels on parallel sections of human tumors. Archival specimens from a cohort of 51 human serous ovarian carcinomas were stained with antibodies specific for Tpr and GANP using immunoperoxidase technique. Examples of immunohistochemical staining patterns seen among the ovarian tumors are shown. The scale bar represents 100 µm. **b** The summary of the scoring categories for the entire cohort of 51 human serous ovarian carcinomas analyzed.

Given that endogenous replication stress and genomic instability are prominent features of cancer^3^, and aberrations of both *TPR* and *GANP* have been linked to diverse types of human malignancies^10,50^, we next assessed the relevance of our results obtained with cell culture models for human clinical tumor specimens. As our model experiments indicated that loss of Tpr affected the abundance of GANP via protein destabilization rather than impacting *GANP* mRNA levels (Supplementary Fig. 5e, f), we chose to approach the clinical relevance of the Tpr–GANP interplay by immunohistochemical analysis of the two proteins on parallel sections of human tumors. Archival specimens from a cohort of 51 human serous ovarian carcinomas were stained with antibodies specific for Tpr and GANP, respectively, using an optimized, sensitive immunoperoxidase technique^55^. Evaluation of Tpr and GANP abundance for each tumor was then performed in detail, scoring the relative levels of either protein in carcinoma cells relative to the surrounding stromal cells as an internal control. Based on our model experiments, our prediction was that in cases with aberrantly low Tpr, GANP would be destabilized and should always follow the decrease. On the other hand, if Tpr would be aberrantly overabundant, GANP might also show an increase. Our analysis showed that in 32 of the 51 tumors, the levels of both Tpr and GANP were similar to levels seen in the stromal cells, and this pattern has been scored as “normal.” In addition, there were 11 cases in which both Tpr and GANP were overabundant (“high”), and 7 tumors with aberrantly low levels of Tpr and GANP (“low” category) relative to stromal cells in the same sections. Examples of immunohistochemical staining patterns seen among the ovarian tumors are presented in Fig. 6a, and the summary of the scoring categories for the entire cohort is provided in Fig. 6b. Overall, among the 51 cases of our cohort, 50 showed concordant results for Tpr and GANP, and in 1 case, GANP level was abnormally low while the level of Tpr remained normal. These results indicate that ~38% of the ovarian carcinomas showed deregulated protein levels of the Tpr–GANP axis. Furthermore, the staining patterns seen in each and every case were consistent with the predictions made from our model experiments, namely that altered levels of Tpr should be followed by a similar change in abundance of GANP, while deregulation of GANP may not impact abundance of Tpr.

Taken together, these results support the notion that the nucleoporin Tpr, along with its interaction partners involved in RNA processing, protects against replication stress. The intimate link between Tpr and GANP is documented by shared phenotypes and patterns seen in human tumors, and Tpr depletion evokes chromosomal instability, one of the hallmarks of cancer.

## Discussion

The nuclear pore complex in general, including the nucleoporin Tpr, may participate in a broader range of cellular functions than so-far appreciated. This concept is consistent with an ever-increasing spectrum of pathologies linked to nucleoporin mutations and/or deregulated expression, including multiple types of common human cancers^8^. Our study was designed to shed more light on the potential role(s) of Tpr in safeguarding genomic integrity, with focus on the interface between DNA replication and transcription processes, an emerging hub of cellular activities requiring stringent regulation under both normal and, particularly, under stressful conditions to maintain and/or restore homeostasis and minimize any harmful consequence of endogenous or environmental insults. The results we obtained through a combination of multiple experimental approaches, cellular models and clinical material, provide insights into this so-far largely unexplored area of cell (patho)biology, in several ways.

First, at the conceptual level, we provide evidence that Tpr is required for cell survival and avoidance of excessive DNA breakage in proliferating human cells. This conclusion reflects our cumulative data from clonogenic assays, monitoring DNA damage signaling, comet assays, and frequency of G1-phase bodies formed by 53BP1, the latter feature commonly used to document the degree of replication stress in the previous cell cycle. Notably, these 53BP1-marked chromatin sites in the G1 daughter cells indicate location of DSBs resulting from broken genomic loci whose replication remained unfinished by the time of previous mitotic entry, i.e., events mechanistically distinct from lagging mitotic chromosomes^4,34^ and therefore not attributable to any defects in the known contribution of Tpr to the mitotic spindle checkpoint^20^. The G1 53BP1 bodies, along with our results demonstrating dependency of the phenotypes on transcription, our data on altered replication fork progression (see below), and the fact that our experiments were largely based on a short pulse of HU-induced replication stress, collectively support a new role of human Tpr in facilitating chromosome replication. Furthermore, our functional experiments show that the observed phenotypes of enhanced replication stress and DNA damage in Tpr-depleted cells depend on ongoing gene transcription, consistent with yeast data^19^, thereby highlighting the emerging intimate links between DNA replication and transcription, and the crucial role of Tpr in coregulating and/or monitoring and modulating the quality of these fundamental nucleic acid transactions, as a concept supported by our study (see “Model” in Supplementary Fig. 7). This overall scenario is also supported by the evidence we provide for enhanced abundance, in a transcription-dependent manner, of DNA–RNA hybrids, RNaseH1-sensitive structures that can accumulate under stressful conditions, and undermine cellular homeostasis including genome integrity^42^.

The second aspect through which our present study contributes to the field is the identification, very stringent selection, and functional annotation of a series of human proteins that interact with Tpr, followed by functional validation of several selected candidates. These results reflect the outcome of three complementary SILAC-based, high-throughput proteomic screening strategies, the integrated results of which are presented in two tables (Table 1; Supplementary Table 1). Furthermore, the data from these experiments allowed us to construct a schematic network of the most significantly enriched Tpr-interacting human proteins, using bioinformatics tools^44^ to further mine our results from the proteomic screens, complemented by information about the functions and biological contexts of the individual Tpr interactors, available from gene and protein databases. The overall outcome that has emerged from these analyses is a concept that places Tpr inside a network of proteins implicated in RNA processing and quality control in human cells. This network encompasses also several proteins implicated in responses to DNA damage, such as the splicing regulator HNRNPC known to interact with prominent DNA repair proteins such as BRCA1 and BRCA2^45^, or the splicing factor MATR3 involved in regulation of residence time of some DDR factors at DNA damage sites, and itself a target of DNA damage checkpoint signaling^30,48^. Most relevant for the concept, we propose here are the functional data demonstrating that depletion of any of the four tested members of the Tpr-interacting network: PTBP1, SUGP2, MATR3 and GANP resulted in replication stress sensitivity phenotypes reminiscent of the responses seen in cells depleted of Tpr itself. Based on these results, we propose that Tpr operates embedded in a protein-interacting network involved in RNA processing and quality control, and that disruption of this pathway by depletion of Tpr or other individual Tpr-associated proteins unmasks a characteristic phenotype of transcription-dependent replication stress, at the same time enhancing sensitivity of human cells to insults that may impair DNA replication.

As the third significant aspect of our current dataset, we wish to emphasize the intimate link between deregulation of proteins in the identified Tpr-interacting network, here directly examined for Tpr and GANP, and human cancer. While nucleoporin aberrations are involved also in pathogenesis of other life-threatening diseases, we chose cancer as a pathological condition in which both Tpr and GANP have been implicated in several ways: through occurrence of fusion oncogenes that involve parts of *Tpr* gene fused with tyrosine kinases such as *Met* or *Trk*, as well as different mutations of *Tpr* or *GANP*, and their deregulated mRNA expression levels found across a wide spectrum of tumors. Another reason to focus on cancer was inspired by the functional links of Tpr with replication stress emerging from our initial experiments. Thus, we and others have previously discovered enhanced endogenous replication stress, attributable to effects of oncogenes or loss of some tumor suppressors, events impacting both transcription and DNA replication, as a common feature shared by many oncogenic models as well as clinical specimens from diverse human malignancies^3,55–57^. Here, we found not only a physical interaction between Tpr and GANP but also evidence for the ability of Tpr to support protein stability and nuclear envelope localization of GANP, an effect that was not reciprocal, as depletion of GANP impacted neither levels nor subcellular localization of Tpr. Notably, our immunohistochemical analysis of Tpr and GANP in a cohort of 51 archival biopsies from human ovarian carcinomas showed not only a sizeable fraction (19) of cases with abnormal levels of Tpr and/or GANP but also confirmed the prediction that whenever Tpr shows either aberrantly reduced protein expression or overabundance in cancer cells, GANP protein always recapitulated these patterns, most likely due to the stoichiometry and stabilizing effects of Tpr on GANP through their interaction, which we found in the cancer cell lines. These results are not only valuable due to the virtual absence of any studies so far of Tpr or GANP in human tumors at the protein level but also as an example of a common human tumor type in which we show Tpr and GANP proteins are frequently deregulated. In the context of our overall results, we speculate that the carcinomas with overabundant Tpr and GANP may benefit from the effect of these proteins in “buffering” the likely enhanced endogenous or potential therapy-induced replication stress, thereby supporting the fitness, survival, and proliferation of those cancers (11 of 51 in our cohort). On the other hand, those tumors featuring the aberrant reduction of Tpr/GANP proteins (8 of 51) may be more susceptible to endogenous oncogenic stress, thus fueling genomic instability, a mechanism known to promote tumor heterogeneity, subclonal evolution, and resistance to standard-of-care treatments^4^. In this context, it may also be meaningful the observation that the BRCA2 tumor suppressor interacts with the PCID2 component of TREX-2, to which GANP belongs^58^.

Finally, our observations show that Tpr-depleted cells exhibit a variety of replication defects: (i) hypersensitivity to DNA synthesis inhibitors; (ii) fork stalling and asymmetry; (iii) unrestrained restart replication events; and (iv) transcription-dependent HU-induced RPA phosphorylation. Notably, we did not observe in Tpr-defective cells fork collapse events such as fork reversal or fork resection. Although other hypotheses can be envisaged to explain our data, we speculate that Tpr deficiency might elicit unscheduled DNA synthesis events triggered by transcription (Supplementary Fig. 7). DNA synthesis primed by transcription has been documented in vivo in bacteria and yeast^59^ and in vitro^60^. Our results indicate that Tpr coordinates at nuclear pores a physical network of enzymatic activities mediating transcription termination, mRNA processing, splicing, and export, thus facilitating the formation of mRNA processing factories (mRPFs) tethering the nuclear envelope and chromatin. These bulky factories may hinder the rotation of the RNA polymerase II complex during transcription, thus generating the ideal conditions for DNA–RNA hybrid formation at the end of the gene^61^ (Supplementary Fig. 7). In Tpr-defective cells, the inefficient mRNA export may further engulf mRNA splicing and processing, thus exacerbating DNA–RNA hybrid formation and even leading to transcription leakage beyond the transcription termination site^61^. These aberrant transcription events, when converging head-on with stalled forks, may unwind the duplex downstream of the forks and merge with the stalled lagging strands. These extended R-loops might give rise to unscheduled leading-strand restart, eventually followed by lagging-strand repriming downstream of the hybrid. A similar aberrant replication context has been described for senataxin-defective cells^62^. This model predicts that head-on collision between transcription and replication forks in Tpr-defective cells would (i) slow down forks, (ii) generate asymmetric forks, (iii) form RPA-ssDNA nucleofilaments in a transcription-dependent manner, and (iv) cause unscheduled repriming of stalled forks. Moreover, none of these events would be associated with fork collapse. Considering that the C-terminal part of GANP can bind and acetylate MCM3 and inhibit replication initiation^53^, while its N-terminal portion has primase activity^54^, it is tempting to speculate that GANP may play a key role in counteracting unscheduled transcription-dependent replication events by coordinating RNA processing. At this stage, however, we cannot rule out potential indirect effects caused by Tpr and GANP depletion, and more work will be required to firmly address the relative contributions of GANP and Tpr in preventing RNA-dependent replication stress. At least part of the phenotypes owing to Tpr depletion could be caused by deregulated transport of RNA or proteins through the nuclear pores, although the role of Tpr in nucleocytoplasmic transport is still not precisely understood^14,22–24^. An important difference between *S. cerevisiae* and human cells is that in yeast mRNA splicing is restrained to few genes, while in human cells most genes undergo splicing. Moreover, while in yeast the Mec1^ATR^ response specifically targets Mlp1^TPR^ to detach transcribed chromatin from the nuclear envelope^18^, it is reasonable to think that in human cells the situation is more complex^63^, and that the ATR pathway coordinates fork advance with transcription by regulating multiple factors operating within mRPFs.

## Methods

### Cell culture and treatments

U-2 OS (ATCC) and HeLa cells (European Collection of Authenticated Cell Cultures) were grown in Dulbecco’s modified Eagle’s medium (Sigma-Aldrich, #D6171) supplemented with fetal bovine serum (Sigma-Aldrich, #F7524) to a final concentration of 10%. BJ cells (ATCC) were grown in minimum essential media (Biowest, #L0440) supplemented with fetal bovine serum (Sigma-Aldrich, #F7524) to a final concentration of 10%. MCF10A cells (ATCC) were grown in Dulbecco’s modified Eagle’s medium/Nutrient Mixture F-12 Ham (Sigma-Aldrich, #D6421) supplemented with horse serum (Thermo Fisher Scientific, #16050122). U-2 OS stable cell line expressing pcDNA4/TO-TPR-2siR-EGFP vector was established according to the manufacturer’s protocol for the creation of stable cell lines using pcDNA4/TO expression vector (Invitrogen). Aph (Sigma, #A0781), cordycepin (Sigma, #C3394), DRB (Sigma-Aldrich, #D1916), HU (Sigma, #H8627), and MG-132 (Sigma, #M7449) treatments were added as indicated.

### Clonogenic cell survival assay

U-2 OS cells were transfected with siRNA targeting Tpr or siRNA control. The next day, cells were plated in a triplicate at three densities depending on the expected survival. Seventy-two hours after the transfection, cells were exposed to Aph or HU for 48 h. After the drug removal, cells were grown for an additional 8 days. Colonies were fixed with Gram’s crystal violet solution (Merck, #1.09218) and scored.

### DNA cloning and transfections

A fully sequenced cDNA clone encoding human Tpr was obtained from Source BioScience (clone OCABo5050B0425D; sequence accession: BC156389). Tpr coding sequence was cloned into pcDNA4/TO vector (Invitrogen, #V1020-20) containing the GFP-tag and made resistant against siRNAs targeting Tpr (Thermo Fisher Scientific, Silencer Select siRNAs ID #s14353 and #s14354) by introducing silent point mutations using the QuikChange XL Site-Directed Mutagenesis Kit (Agilent Technologies, #200517) resulting in pcDNA4/TO-TPR-2siR-EGFP vector. The oligonucleotides used for mutagenesis in this study are presented in Supplementary Table 2. Lipofectamine 2000 (Thermo Fisher Scientific, # 11668019) was used for the transfection of pcDNA4/TO-TPR-2siR-EGFP, pcDNA4/TO-GFP, Tpr-FLAG, Tpr-C, and Tpr-N vectors, and pEGFP-N2 (Clontech) or pEGFP-M27-H1^64^ plasmids.

siRNA transfections were performed using Lipofectamine RNAiMAX Transfection Reagent (Thermo Fisher Scientific, #13778075) or DharmaFECT 1 (Horizon Discovery) according to the manufacturer’s manual. siRNAs were used at the final concentration of 25 nM (Thermo Fisher Scientific) or 100 nM (Horizon Discovery). All assays were performed 72 h after siRNA transfection and 24 h after plasmid transfection, except for alkaline and neutral comet assays, which were performed 48 h after siRNA transfection. The oligonucleotides used for individual assays in this study are presented in Supplementary Table 2.

### DNA combing

DNA combing was performed as described^65^. Briefly, cells were pulse-labeled with 25 μM iododeoxyuridine (IdU) for 20 min and with 200 μM chlorodeoxyuridine (CldU) for 20 min. Anti-ssDNA from Developmental Studies Hybridoma Bank was used instead of the one described. Fork Symmetry was calculated as IdU/CldU ratio. Fork Velocity was calculated as reported^66^. Images were acquired with a Leica DM6000 microscope equipped with a DFC390 camera (Leica) at 40× magnification. Metamorph software (version v7.5.1.0) (Molecular Probes) was used for region measurements.

### DNA fiber analysis

In the experiment analyzing replication fork progress in unperturbed conditions, U-2 OS cells transfected with siTPR or siControl were pulse-labeled with 25 μM CldU (Sigma-Aldrich, #C6891) for 20 min, gently washed (twice with prewarmed 1xPBS and once with DMEM) and pulse-labeled with 250 μM IdU (Sigma-Aldrich, #I7125) for 20 min.

In the experimental setup evaluating stop of the replication fork under the conditions of the replication stress, siRNA transfected cells were pulse-labeled with 25 μM CldU for 20 min, gently washed, and treated with 2 mM HU for 4 h. Twenty minutes before the end of the HU treatment, 250 μM IdU was added directly to the medium, and cells were pulse-labeled for 20 min.

To analyze the replication fork restart, siRNA transfected cells were pulse-labeled with 25 μM CldU for 20 min, gently washed, and treated with 2 mM HU for 4 h. Following HU treatment, cells were gently washed and pulse-labeled with 250 μM IdU for 20 min without HU.

Pulse-labeled cells were collected, and DNA fiber spreads were prepared as described previously^67^. In brief, for all experimental conditions, five slides were stretched, and 2–3 slides for each condition were stained. CldU was detected using rat anti-BrdU antibody (Bio-Rad, #OBT0030, 1:1000) for 1 h. Slides were fixed 10 min with 4% PFA and incubated with goat anti-rat IgG Alexa Fluor 568 (Thermo Fisher Scientific, #A-11077, 1:500) for 1.5 h. IdU was detected using mouse anti-BrdU antibody (BD Biosciences, #347580, 1:1000) overnight at 4 °C and donkey anti-mouse IgG Alexa Fluor 488 (Thermo Fisher Scientific, #A-21202, 1:500) for 1.5 h. Samples were mounted into ProLong Gold Antifade Mountant (Thermo Fisher Scientific, #P36930).

Images of well-spread DNA fibers were acquired using an LSM700 confocal microscope (Carl Zeiss) and a Plan-Apochromat 63×/1.4 numerical aperture (NA) oil immersion objective (Carl Zeiss). Semiautomatic acquisition by using software autofocus and tile-arrays was used to acquire images. Double-labeled replication forks were analyzed manually using ZEN lite 2012 (Carl Zeiss) software. Overall, 50–100 forks were scored for each slide, and fork measurements were pooled together for each experiment.

### DNA–RNA hybrid immunoprecipitation

DRIP was performed as previously described^68^. Briefly, cells were treated with SDS and proteinase K, then DNA was carefully extracted with phenol:chloroform:isoamyl alcohol (24:24:1) followed by isopropanol precipitation. Precipitated DNA was spooled on a glass rod, washed twice with 70% ethanol, gently resuspended in 1xTE, and enzymatically digested with HindIII, EcoRI, BsrGI, XbaI, and SspI in an appropriate commercial buffer. Samples were split and treated with *E. coli* RNase H (NEB) or mock-treated. DNA–RNA hybrids immunoprecipitation was performed by overnight incubation with Protein A-Dynabeads (Invitrogen) coated with the S9.6 antibody at 4 °C using 1x binding buffer (100 mM NaPO4, pH 7.0, 1.4 M NaCl, 0.5% Triton X-100). DNA was eluted with elution buffer (50 mM Tris, pH 8.0, 10 mM EDTA, 0.5% SDS), treated with proteinase K, and purified with the MACHEREY-NAGEL DNA kit. Real-time qPCR was performed at the indicated regions. S9.6 signal was determined by dividing the immunoprecipitated signal to the input for each sample. The relative abundance of DNA–RNA hybrid immunoprecipitated in each region was normalized to the signal of the siRNA control sample without RNase H treatment, when indicated.

### EM analysis of genomic DNA

Analysis of in vivo DNA RIs by the combination of psoralen cross-linking and EM has been conducted as previously reported^69^ with some modifications indicated in the next paragraphs.

### In vivo psoralen cross-linking

Every sample corresponded to 2 × 10^7^ U-2 OS cells in total growing on four 150 × 20 mm tissue culture dishes with 80% confluency. Cells were trypsinized, collected into 40 ml of DMEM, transferred to 50 ml Falcon tubes, and centrifugated at 290 × *g* for 5 min. The cell pellet was washed once (by resuspension/centrifugation) with 5 ml of ice-cold 1xPBS.

Cells were resuspended in 3 ml of ice-cold 1xPBS and transferred into a tissue culture dish 60 × 15 mm. Fifty microliters of 4,5′,8-trimethylpsoralen dissolved in DMSO (Sigma-Aldrich, #T6137, 2 mg/ml stock solution) was added to the cell suspension to a final concentration 33 μg/ml. After 5 min mixing on the magnetic stirrer on ice and in the dark, samples were irradiated for 5 min using a Stratalinker UV Crosslinker Model 1800 (Stratagene, #400072) equipped with UVA 365 nm lamp. Addition of the fresh the 4,5′,8-trimethylpsoralen and incubation in the dark under stirring followed by 5 min cross-linking cycle under UVA were repeated three more times for a total of four cross-linking cycles.

The cell suspension was transferred to a 15 ml Falcon tube and centrifugated at 290 × *g* for 5 min. The cell pellet was resuspended in 3 ml of TNE buffer (10 mM Tris-Cl, pH 7.4, 100 mM NaCl, 10 mM EDTA). Ten microliters of RNase A (Sigma-Aldrich, #R5000, 10 mg/ml stock solution) to a final concentration of 16 μg/ml and 3 ml of TNES buffer (10 mM Tris-Cl, pH 7.4, 100 mM NaCl, 10 mM EDTA, 1% SDS) were added. Samples were placed to a water bath at 37 °C for 1 h. Next, 50 μl of proteinase K (Roche, #3115887001, stock solution 10 mg/ml) was added to a final concentration of 83 μg/ml, and samples were incubated without shaking overnight in a water bath at 37 °C.

### DNA extraction

We did not use ionic exchange columns to purify the genomic DNA analyzed in this study. Instead, the genomic DNA was isolated using phenol-chloroform extraction. Six milliliters of phenol:chloroform:isoamyl alcohol 25:24:1 (Sigma-Aldrich, #P2069) was added to the samples after the overnight incubation and samples were mixed by reverting the Falcon tubes for 1 min. Next, samples were centrifugated at 3200 × *g* at room temperature for 5 min, and 5 ml of upper phase containing DNA was transferred to a new 15 ml Falcon tube. Five milliliters of chloroform (VWR Chemicals, #22711.230) was added, samples were mixed by reverting the Falcon tubes for 1 min, and centrifugated at 3200 × *g* at room temperature for 5 min. Five milliliters of the upper phase was collected and moved to a new 15 ml Falcon tube. Five hundred microliters of 3 M sodium acetate, pH 5.2, was added. After the addition of 5.5 ml of isopropanol (VWR, #20842.330), precipitated DNA was transferred using pipette with 200 μl tip to the Eppendorf tube with 70% ethanol (VWR, #20821.330) and centrifugated at 16,000 × *g* at 4 °C for 3 min. Finally, DNA pellet was dried at room temperature for 15 min and resuspended in 300 μl of 10 mM Tris-HCl, pH 8.0.

### BND-cellulose enrichment

For the enrichment of the DNA RIs, we used BND cellulose as described previously^69^. In brief, 24 μg of genomic DNA was digested with PvuII-HF (New England Biolabs, #R3151S) for 5 h at 37° C. Following DNA enzymatic digestion, the digestion mix was adjusted to 300 mM NaCl final concentration by adding 5 M NaCl stock. The final volume was adjusted to 1200 μl with 10 mM Tris-HCl, pH 8.0, 300 mM NaCl. One milliliter of BND cellulose (Sigma-Aldrich, #B6385) was applied to Poly-Prep Chromatography Columns (Bio-Rad, # 7311550). Columns were washed six times with 1 ml of 10 mM Tris-HCl, pH 8.0, 1 M NaCl and equilibrated six times with 1 ml of 10 mM Tris-HCl, pH 8.0, 300 mM NaCl. Next, DNA digestion mix was loaded on BND cellulose and incubated at room temperature for 30 min. To allow binding of DNA molecules to the BND cellulose, BND-resin bed was gently resuspended every 5 min. After the incubation, the columns were washed twice with 1 ml of 10 mM Tris-HCl, pH 8.0, 1 M NaCl. In the next step, 600 μl of 10 mM Tris-HCl, pH 8.0, 1 M NaCl, 1.8% caffeine was applied on BND cellulose. After 5 min, BND cellulose was gently resuspended and incubated for an additional 5 min. The caffeine fraction, enriched in DNA RIs, was collected at the end of the incubation.

DNA was loaded on Amicon conical filters 100k (0.5 ml) (Millipore, #C82301) and centrifugated for 10 min at 5000 × *g*. Next, Amicon filters were washed twice with 500 μl of Tris-HCl 10 mM, pH 8.0 and centrifugated after every wash at 5000 × *g* for 10 min. Finally, DNA samples were transferred to fresh tubes according to the manufacturer’s conditions.

### DNA spreading

EM samples were prepared using the digested genomic DNA enriched on BND-cellulose columns. Briefly, the formamide/BAC spreading protocol was used to create a monomolecular layer of DNA molecules on the surface of the water. The monomolecular layer of DNA molecules was touched with a carbon coated copper grid (4–5 nm of carbon without support). Absorbed DNA molecules on the carbon surface were positively stained with uranyl acetate resuspended in ethanol and dried girds with absorbed DNA molecules were subjected to low angle rotary shadowing with 8 nm of platinum under deep vacuum as described previously^69^. Images of the metallic replica of the positively stained DNA molecules were acquired with a FEI Tecnai12 microscope run at 120 KV with a side-mounted Gatan Orius SC-1000 camera controlled by the Gatan Microscopy Suite Software (Gatan, version 2.3.3 64 bit). Image files were saved in the dm3 format of digital micrograph and analyzed with ImageJ software (version 2.1.0/1.53C). In these conditions, the thickness of the double strand DNA fiber is distributed around 10 nm, and single-strand DNA fibers (or stretches) have a thickness, which is half of the double strand DNA thickness^69,70^. In these conditions, the conversion factor for the measurements of the length of the dsDNA fibers was 0.36 nm/bp^70^.

### Flow cytometry

U-2 OS cells were labeled with 10 µM EdU for 20 min. Next, cells were fixed using 3.6% PFA in 1xPBS for 15 min and permeabilized with 0.25% Triton X-100 for 15 min at room temperature. *S* phase cells were detected using Click-iT EdU Alexa Fluor 488 Imaging Kit (Invitrogen, #C10337) according to the manufacturer’s protocol, and total DNA content was stained using propidium iodide (2.5 μg/ml) in the presence of RNase A (250 μg/ml). Samples were acquired on a FACS Attune NxT Flow Cytometer (Thermo Fisher Scientific) equipped with Attune Nxt software. Samples were analyzed using FLOWJO 10 software.

### Immunoblotting

Whole cells extracts were prepared using 1x Laemmli sample buffer (2% SDS, 10% glycerol, 50 mM Tris-HCl, pH 6.8). Cell lysates were sonicated (Bandelin Sonoplus HD 2070, Bandelin, Germany) and centrifugated at 16,000 × *g* at 4 °C for 30 min. Total protein concentration in each sample was determined using BCA protein assay (Thermo Fisher Scientific, #23225). Proteins were resolved by SDS-PAGE and transferred onto nitrocellulose membranes. Membranes were blocked using PBS with Tween 20 (0.02%) containing 5% milk powder at room temperature for 30 min. Primary antibodies were diluted in 1xPBS with Tween 20 (0.02%) containing 1% milk and applied overnight. Mouse or rabbit secondary antibodies were diluted in 1xPBS with Tween 20 (0.02%) containing 1% milk powder and applied for 1 h at room temperature prior to detection using a SuperSignal West Dura Extended Duration Substrate (Thermo Fisher Scientific, #34076). The following primary antibodies were used: ATR (Cell Signaling, #2790, 1:1000), phospho-ATR (Thr1989) (Cell Signaling, #58014, 1:1000), Chk1 (Santa Cruz Biotechnology, #sc-8408, 1:1000), phospho-Chk1 (Ser345) (Cell Signaling, #2348, 1:1000), phospho-Chk1 (Ser317) (Cell Signaling, #12302, 1:1000), cyclin A2 (Abcam, #ab16726, 1:000), GANP (Abcam, #ab113295, 1:1000), GANP (Sigma-Aldrich, #HPA021527, 1:1000), GFP (Amsbio, TP401, 1:2500), MATR3 (Bethyl, #A300-591A, 1:1000), PTBP1 (Thermo Fisher Scientific, #32-4800, 1:000), RPA32 (Abcam, #ab16855, 1:2000), phospho-RPA32 (S4/S8) (Bethyl, #A300-245A, 1:1000), SUGP2 (Sigma-Aldrich, #HPA061111, 1:1000), Tpr (Sigma-Aldrich, #HPA019661, 1:1000), and tubulin (Sigma-Aldrich, #T5168, 1:5000). The following secondary antibodies were used: goat anti-mouse IgG (H+L)-HRP conjugate (Bio-Rad, #1706516, 1:5000) and goat anti-rabbit IgG (H+L)-HRP conjugate (Bio-Rad, #1706515, 1:5000).

### Immunostaining

Cells growing on 12 mm coverslips were fixed using 3.6% formaldehyde (Sigma, #47608) in 1xPBS for 15 min at room temperature, washed once with 1xPBS, and permeabilized at room temperature in 0.2% Triton X-100 (Biochemica, EMR237500) in 1xPBS for 15 min. Next, coverslips were washed twice with 1xPBS and blocked with 1xPBS containing filtered 10% fetal bovine serum (Sigma-Aldrich, #F7524) at room temperature for 30 min. Primary and secondary antibodies were diluted in 1xPBS containing filtered 10% fetal bovine serum (Sigma-Aldrich, #F7524). Coverslips were incubated with the primary antibodies at room temperature for 1 h, washed three times with 1xPBS for 5 min, and incubated with the secondary antibodies at room temperature for 1 h. Next, coverslips were washed three times with 1xPBS for 5 min and mounted on 4 μl Vectashield antifade mounting medium with DAPI (Vector Laboratories, #H-1200).

For γH2AX and 53BP1 foci analysis in HeLa cells, cells were fixed in 2% formaldehyde in 1xPBS for 20 min at room temperature and permeabilized with 70% ethanol at −20 °C for 5 min, at 4 °C for 5 min, and washed twice in 1xPBS. After blocking with 1xPBS containing 3% bovine serum albumin (BSA), the coverslips were incubated with primary antibodies diluted in 1xPBS containing 3% BSA at RT for 2 h. Then cells were washed three times in 1xPBS for 5 min. Secondary antibodies conjugated with Alexa Fluor diluted (1:1000) in 1xPBS containing 3% BSA were incubated at room temperature for 1 h. Coverslips were washed twice in 1xPBS before and after the staining of the DNA with 1 μg/ml DAPI for 5 min. Finally, coverslips were washed in water and a drop of Immu-Mount mounting medium (Thermo Fisher Scientific) was used for mounting.

For S9.6 and nucleolin immunofluorescence, cells were fixed in cold absolute methanol at −20 °C for 7 min and washed twice in 1xPBS. Coverslips were blocked in 1xPBS containing 2% BSA at 4 °C overnight, incubated with anti-S9.6 and anti-nucleolin primary antibodies diluted in 1xPBS containing 2% BSA at 4 °C overnight, washed in 1xPBS, and incubated with secondary antibodies conjugated with Alexa Fluor diluted in 1xPBS containing 2% BSA (1:1000) at room temperature for 1 h. Washes, DAPI staining, and mounting were performed as described above. The following primary antibodies were used: cyclin A2 (Abcam, #ab16726, 1:200), Flag M2 (Sigma-Aldrich, #F1804, 1:100), GANP (Sigma-Aldrich, #HPA021527, 1:100), phospho-H2A.X (Ser139) (Millipore JBW301, #05-636, 1:500), nucleolin (Abcam, #ab50279, 1:1000), S9.6 (Hybridoma cell line HB-8730, 1:100), Tpr (Sigma-Aldrich, #HPA019661, 1:100), and 53BP1 (Novus Biologicals, #NB100-304, 1:500).

The following secondary antibodies were used:

Alexa Fluor 488 (Thermo Fisher Scientific, #A-21206, 1:400), Alexa Fluor 488 (Molecular Probes, #A11008 and #A11029, 1:1000), Alexa Fluor 546 (Molecular Probes, #A21123, 1:1000), Alexa Fluor 568 (Molecular Probes, #A11011, 1:1000), Alexa Fluor 594 (Molecular Probes, #A21201, 1:1000), Alexa Fluor 647 (Molecular Probes, #A21244 and #A21463, 1:1000), and Cy3 (Jackson ImmunoResearch Laboratories, #715-165-150, 1:400).

### Immunohistochemical analysis

Five-micrometer tissue sections were cut from the formalin-fixed, paraffin-embedded tissue blocks and mounted on Super Frost Plus slides (Menzel-Gläser, Braunschweig, Germany), baked at 60 °C for 60 min, deparaffinized, and rehydrated through graded alcohol rinses. Heat induced antigen retrieval was performed by immersing the slides in citrate buffer (pH 6.0) and heating them in a microwave oven for 15 min, followed by immunohistochemistry staining with the primary antibodies as follows: anti-Tpr antibodies (Sigma-Aldrich, #HPA019661, diluted 1:1000 and Sigma-Aldrich, #HPA024336, diluted 1:250) and anti-GANP antibody (Sigma-Aldrich, #HPA021527, diluted 1:250). The sections were incubated with the primary antibodies overnight in a cold-room, followed by subsequent processing by the indirect streptavidin-biotin-peroxidase method using the Vectastain Elite kit (Vector Laboratories, Burlingame, CA, USA) and nickel-sulfate-based chromogen enhancement detection as previously described, without nuclear counterstaining^55^. For negative controls, sections were incubated with nonimmune sera. The results were evaluated by two experienced researchers, including a senior oncopathologist, and the data scored in three categories, expressed as percentage of cases in which: (i) the intensity of the staining signal was similar in the cancer cells as in the surrounding stromal cells (“normal” pattern), (ii) the staining was clearly more intense in the cancer cells than in the stromal cells (“high”), and (iii) cases where the tumor cells were less positive compared with the stromal cells (“low” category).

The cohort of archival formalin-fixed, paraffin-embedded tissues from human serous carcinomas of the ovary (*n* = 51) was described recently^71^. This archival material from the ovarian tumors was collected at the Department of Pathology, University Hospital, Las Palmas, Gran Canaria, Spain, from surgical operations performed in the period 1995–2005. For the purpose of the present study, only samples from serous carcinomas (the most common type of ovarian malignancy) were used. This study was undertaken in accordance with the Spanish codes of conduct (Ley de Investigación Biomédica) and with the World Medical Association Declaration of Helsinki, after approval by the review board of the participating institution: the University Hospital, Las Palmas, Gran Canaria, Spain. Patients were informed that samples may be used for research purposes under the premise of anonymity.

### Immunoprecipitation

For the immunoprecipitation, previously published protocol was used^72^. In brief, cells growing on 145 mm dishes were washed twice with a cold 1xPBS, harvested and centrifugated at 290 × *g* at 4 °C for 5 min. Next, cells were resuspended in ice-cold lysis buffer containing 20 mM Tris at pH 8.0, 150 mM NaCl, 0.5% NP40, 1 mM EDTA, 1 mM MgCl2, supplemented with the protease (Roche, #05892791001), and phosphatase (Roche, #04906837001) inhibitor cocktail tablets. Benzonase nuclease (Merck, #70664) was added to the final concentration of 50 units/ml to digest all forms of DNA and RNA to eliminate nonspecific interactions bridged by DNA and/or RNA. Cells were incubated on ice for 90 min with pipetting every 30 min. Next, cell lysates were centrifugated at 14,000 × *g* at 4 °C for 15 min. The lysates were transferred to precooled tubes, and the reaction volume was adjusted with the dilution buffer (the same composition as the lysis buffer without benzonase nuclease). Lysates were precleared with immunoprecipitation beads at 4 °C for 2 h. After the preclearing, lysates were centrifugated at 2500 × *g* at 4 °C for 2 min and transferred to new falcon tubes.

Depending on the experimental setup, either GFP-Trap Agarose beads (Chromotek, #gta-20) or recombinant Protein G-Sepharose 4B beads (Thermo Fisher Scientific, #10-1242) coupled with antibodies were used for the overnight immunoprecipitation performed at 4 °C on lysates normalized by protein concentration. Next day, lysates with the beads were centrifugated at 2500 × *g* at 4 °C for 2 min. Supernatants were discarded, beads were washed three times with a dilution buffer and resuspended in 2x SDS Sample Loading Buffer (4% SDS, 20% glycerol, 100 mM Tris-HCl, pH 6.8). Protein elution was performed by heating the samples at 70 °C for 10 min.

### Microscopy and image analysis

Images were acquired with a DM600B fluorescence microscope (Leica Microsystems, Germany), confocal images were acquired on a Leica TCS SP5 or SP8 system (Leica Microsystems, Germany) equipped with a DMi8 inverted microscope using HC PL APO CS2 63×/1.40 oil immersion objective. For the excitation, a white light laser tuned at 488 nm (for Alexa 488) and 561 nm (for Cy3) was used. For the DAPI acquisition, 405 nm laser line was used. Images were acquired as single z sections with an optical zoom of 2× and a format of 2048 × 2048 pixels. The software used was the Leica Application Suite X (version 3.5.2.18963). Images were processed using Fiji software (version 1.52u)^73^ and smoothed to reduce the background noise.

For a quantitative analysis of 53BP1 foci in U-2 OS cells, a series of nonoverlapping frames were randomly acquired using an Olympus IX81 widefield microscope driven by ScanR software (version 2.3.0.7) and equipped with a 40× oil immersion objective (1.00 NA). More than 100 fields per sample were automatically acquired for each condition (about 1000 cells analyzed/slide). Image analysis was performed with the image-analysis software CellProfiler (version 3.1.8)^74^. Briefly, DAPI and cyclin A channels were used to identify nuclei and to discriminate the G1 and S/G2 cell cycle phases, respectively. Foci were then recognized in the nuclear regions in parallel in these two populations of cells, and their number was evaluated. An enhancement filter was applied to the foci channel in order to reduce the background noise and to improve foci recognition. GraphPad Prism software (version 8.4.3.686) was used for all statistical analyses.

### Real-time PCR

U-2 OS cells were transfected using siRNAs for 72 h. RNA was purified using the RNeasy Mini Kit (Qiagen, #74104) according to the manufacturer’s protocol. Reverse transcription of total RNA to cDNA was performed using High-Capacity cDNA Reverse Transcription Kit (Applied Biosystems, #4368814) according to the manufacturer’s protocol. LightCycler 480 SYBR Green I Master reaction mix (Roche, #04707516001) was used for the real-time PCR using the LightCycler 96 Instrument (Roche, #05815916001) according to the manufacturer’s protocol. Relative quantification analysis was used to compare the target gene of interest (GANP) and the reference gene (GAPDH) and to express the final result as a ratio of these genes. LightCycler 96 software (Roche, version 1.1.0.1320) was used for the analysis following the LightCycler 96 System User Training Guide V2.0.

### SILAC and mass spectrometry

Three independent experimental setups were used to improve the accuracy of our results. In the first experimental setup, cells were cultured in DMEM for SILAC medium (Thermo Fisher Scientific, #89985) containing light- (^12^C_6_,^14^N_4_–Arg, ^12^C_6_,^14^N_2_–Lys) (Sigma, #L5751, #A6969) or medium-labeled (^13^C_6_-Arg, ^4^D-Lys) (Cambridge Isotope Laboratories, #CLM-2265-H, #DLM-2640-0) amino acids. Four days after the start of amino acids labeling, cells were transfected with siRNA targeting endogenous Tpr (Thermo Fisher Scientific, Silencer Select siRNA ID #s14353) using Lipofectamine RNAiMAX Transfection Reagent (Thermo Fisher Scientific, #13778075). Five days after the start of amino acids labeling, cells growing in the medium with light-labeled amino acids were transfected with the vector expressing a siRNA-resistant version of Tpr (pcDNA4/TO-TPR-2siR-EGFP) using Lipofectamine 2000 (Thermo Fisher Scientific, # 11668019). In parallel, the cells growing in DMEM medium containing medium-labeled amino acids were transfected with empty vector (pcDNA4/TO-GFP), serving as a negative control for the immunoprecipitation. At day 7, cells were harvested, and Tpr-GFP and GFP were immunoprecipitated using the GFP-Trap Agarose beads (Chromotek, # gta-20).

In the second experimental setup, cells were cultured in DMEM for SILAC medium (Thermo Fisher Scientific, #88364) containing light- (^12^C_6_,^14^N_4_–Arg, ^12^C_6_,^14^N_2_–Lys) (Sigma, #L5751, #A6969) or heavy-labeled (^13^C_6_,^15^N_4_–Arg, ^13^C_6_,^15^N_2_–Lys) (Cambridge Isotope Laboratories, #CNLM-539-H, #CNLM-291-H) amino acids. At day 5 after the start of amino acids labeling, the cells growing in a medium with light-labeled amino acids were transfected with siRNA control (Thermo Fisher Scientific, Silencer Select Negative Control #1). In parallel, cells growing in a medium containing heavy-labeled amino acids were transfected with siRNA targeting Tpr (Thermo Fisher Scientific, Silencer Select siRNA ID #s14353), serving as a negative control for the immunoprecipitation. At day 8, cells were harvested, and endogenous Tpr was immunoprecipitated using anti-Tpr antibody (Sigma-Aldrich, #HPA019661) coupled with the rec-Protein G-Sepharose 4B Conjugate beads (Thermo Fisher Scientific, # 10-1243).

In the last experimental setup, cells were growing in the DMEM for SILAC medium (Thermo Fisher Scientific, #88364) containing light- (^12^C_6_,^14^N_4_–Arg, ^12^C_6_,^14^N_2_–Lys) (Sigma, #L5751, #A6969) or heavy-labeled (^13^C_6_,^15^N_4_–Arg, ^13^C_6_,^15^N_2_–Lys) (Cambridge Isotope Laboratories, #CNLM-539-H, #CNLM-291-H) amino acids. Ten days after the start of the labeling with the amino acids, endogenous Tpr was immunoprecipitated using anti-Tpr antibody (Sigma-Aldrich, #HPA019661) coupled with the rec-Protein G-Sepharose 4B Conjugate beads (Thermo Fisher Scientific, # 10-1243) from the cell growing in DMEM medium containing light-labeled amino acids. In parallel, cells growing in DMEM medium with the heavy-labeled amino acids were subjected to control immunoprecipitation using IgG antibody (Santa Cruz Biotechnology, #sc-2027), serving as a negative control for the immunoprecipitation.

In all the experiments, beads used for immunoprecipitation were washed and pooled before elution with 2x Laemmli sample buffer (4% SDS, 20% glycerol, 100 mM Tris-HCl, pH 6.8) by heating the samples at 70 °C for 10 min. Proteins were then resolved onto a 4–12% Bis-Tris precast gel (Thermo Fisher Scientific, #NW04125BOX) and stained by Coomassie colloidal blue. The gel lane was cut into 8–10 slices, each of which was reduced, alkylated, and digested with trypsin as reported elsewhere^75^. Peptides mixtures were desalted and concentrated on a home-made C_18_ desalting tip, and peptides were injected in a nano HPLC EasyLC (Proxeon Biosystems, Denmark). Peptides separation occurred onto a 25 cm long column, reverse phase spraying fused silica capillary column (75 μm i.d.) packed in house with 3μm ReproSil AQ C18 (Dr. Maisch GmbH, Germany) resin. A gradient of eluents A (HPLC-grade water with 0.1% v/v formic acid) and B (ACN with 20% v/v water with 0.1% v/v formic acid) was used to achieve separation, from 7 to 60% of B in 30 min, at a constant flow rate of 250 nl/min. The LC system was connected to a Q Exactive-HF mass spectrometer (Thermo Fisher Scientific, Germany) equipped with a nanoelectrospray ion source (Proxeon Biosystems, Denmark).

Full scan mass spectra were acquired in the LTQ Orbitrap mass spectrometer with the resolution set to 60,000 (@200 *m*/*z*) accumulating ions to a target value of 3,000,000. The acquisition mass range for each sample was from *m*/*z* 300 to 1650 Da and the analyses were made in technical duplicates. The 15 most intense doubly and triply charged ions were automatically selected and fragmented in the ion trap after accumulation to a “target value” of 15,000. Target ions already selected for the MS/MS were dynamically excluded for 20 s. Identification and quantification of peptides and proteins were performed with MaxQuant (version 1.5.2.8) against the human Uniprot complete proteome set. Criteria for identification of a protein were: at least two peptides (one unique) sequenced, six amino acids of minimal peptide length, FDR < 1%, and quantification achieved with at least two ratio counts. The same dataset was searched against a database for potential contaminants, provided by the software developers, and against a reverse sequence database generated by MaxQuant itself. Significant outliers scores were calculated using Perseus (version 1.5.2.6)^76^ and those with *p* value < 0.05 were selected for further analysis. Pathway analysis was performed using the STRING (version 11.0, http://string-db.org/) and Enrichr (http://amp.pharm.mssm.edu/Enrichr). Pathways with the highest probability score and the highest number of hits have been further considered.

### Single-cell gel electrophoresis (comet assay)

Comet assay was performed using a commercial kit (Trevigen, Gaithersburg, MD, USA) following the manufacturer’s protocol, 48 h after siRNA transfection. When indicated, RNaseH1 was overexpressed, or 50 μM cordycepin was added to the culture 4 h before the experiment. Comet assay with RNaseH1 or cordycepin was performed 72 h after transfection. More than 100 cells derived from three independent experiments were scored.

#### Alkaline comet assay

Cells were collected using accutase, washed and resuspended in ice-cold 1xPBS, combined with low melting agarose, immobilized on CometSlides (30 min at 4 °C), and lysed for 30 min at 4 °C. Then, DNA was unwound and denatured in freshly prepared alkaline unwinding solution (200 mM NaOH, 1 mM EDTA, pH > 13) for 30 min at room temperature, and electrophoresis was performed in prechilled alkaline electrophoresis solution (200 mM NaOH, 1 mM EDTA, pH > 13) at 21 V for 30 min. Next, slides were immersed twice in dH2O for 5 min each, then in 70% ethanol for 5 min and dried at room temperature. DNA was stained with SYBR Green at 4 °C for 5 min.

#### Neutral comet assay

Cells were collected and immobilized on CometSlides as in alkaline comet assay. Cells were lysed for 1 h at 4 °C and immersed in prechilled 1x neutral electrophoresis buffer (for 500 ml, 60.57 g Tris Base and 204.12 g of sodium acetate were dissolved in H2O and the pH adjusted to 9.0 with glacial acetic acid) for 30 min at 4 °C. Electrophoresis was performed in prechilled 1x neutral electrophoresis buffer at 35 V for 15 min and then immersed in DNA precipitation solution (1 M NH4Ac in 70% ethanol) for 30 min at RT. Finally, slides were immersed in 70% ethanol for 30 min at RT and dried. DNA was stained with SYBR Green at 4 °C for 30 min.

### Reporting summary

Further information on research design is available in the Nature Research Reporting Summary linked to this article.

## Supplementary information

Supplementary Information

Reporting Summary

## Data Availability

Source data are provided with this paper and all data are available from the authors upon reasonable requests. The datasets underlying proteomic analysis generated and analyzed during the current study are available in the Peptide Atlas repository. Dataset Identifier: PASS01419. Dataset URL: http://www.peptideatlas.org/PASS/PASS01419.

## Source data

Source Data
