# Supplementary material for: The human nucleoporin Tpr protects cells from RNA-mediated replication stress
Source: Nat Commun. 2021 Jun 24;12:3937. doi: 10.1038/s41467-021-24224-3 (PMC8225803; doi:10.1038/s41467-021-24224-3)
Supplement: Supplementary file 1 — Supplementary Information [file 41467_2021_24224_MOESM1_ESM.pdf]

The human nucleoporin Tpr protects cells from RNA-mediated replication stress

Kosar et al., Nature Communications 2021

Supplementary Information

Supplementary Figures 1-7

Supplementary Tables 1-2

Supplementary Figures

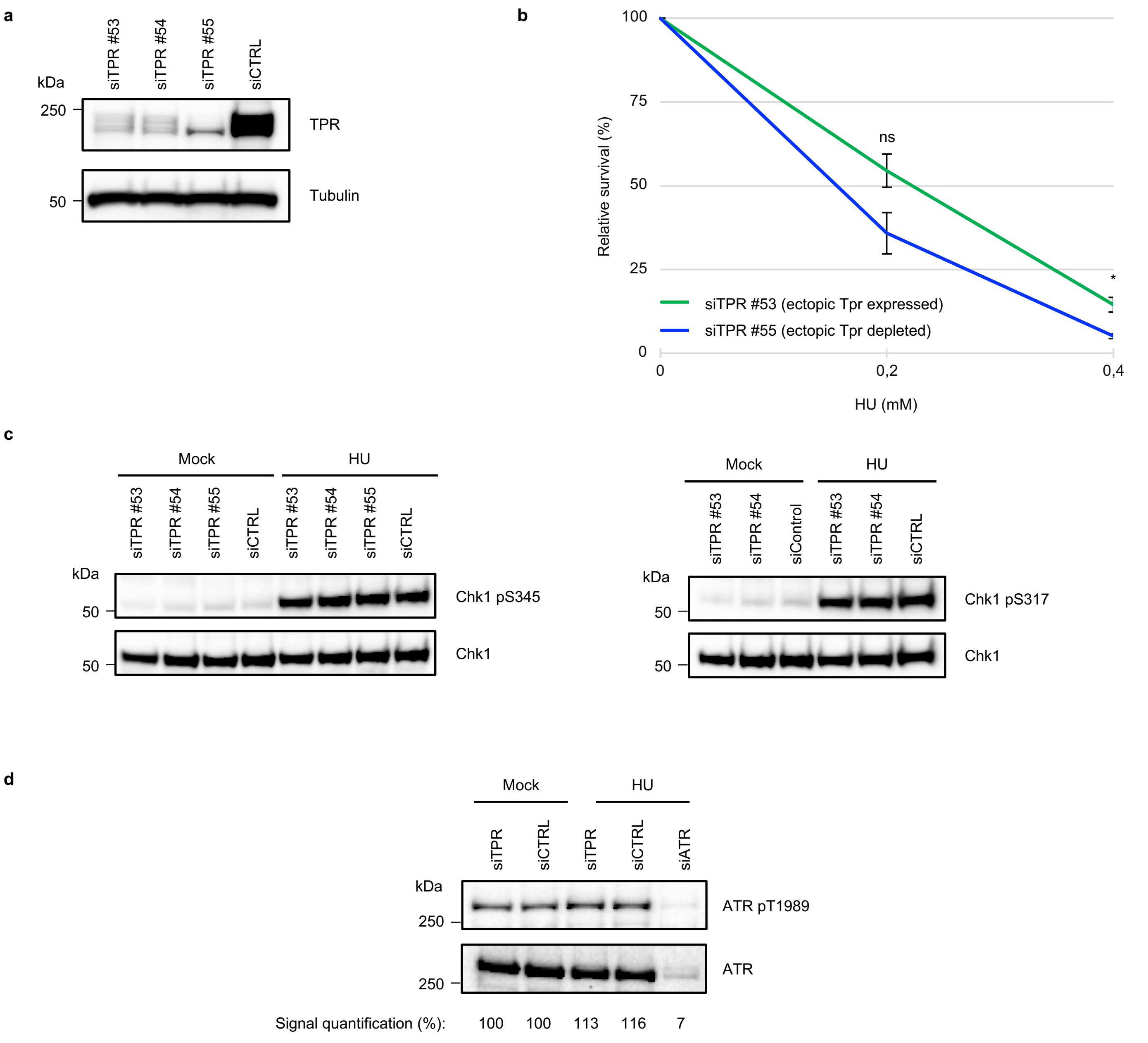

Supplementary Fig. 1 Tpr depletion sensitizes cells to replication stress. a Validation of three different siRNA targeting Tpr (siTPR #53, siTPR #54, siTPR #55) using Western blotting. Tubulin was used as a loading control. The experiment was performed twice yielding similar results. **b** Rescue of the clonogenic survival of U-2 OS cells stably expressing siTPR #53-insensitive wild-type Tpr treated with hydroxyurea (HU). Cells were transiently transfected with siTPR #53 (targeting only endogenous Tpr) or siTPR #55 (targeting both endogenous Tpr and siTPR #53-insensitive Tpr). Data are represented as mean ± SEM from three independent experiments (n=3). Statistical analysis: Welch's t-test; ns not significant; * p-value<0.05. **c** Left: Analysis of Chk1 pS345 phosphorylation in Tpr-depleted cells. Cells were transfected with three independent siRNAs targeting Tpr (siTPR #53, siTPR #54, siTPR #55) or control siRNA (siCTRL). Transfected cells were either non-treated (Mock) or treated with 2 mM hydroxyurea (HU) for 4 hours. Chk1 total was used as a loading control. Right: Analysis of Chk1 pS317 phosphorylation in Tpr-depleted cells. Cells were transfected with two independent siRNAs targeting Tpr (siTPR #53 and siTPR #54) or control siRNA (siCTRL). Transfected cells were either non-treated (Mock) or treated with 2 mM hydroxyurea (HU) for 4 hours. Chk1 total was used as a loading control. Both experiments were performed twice yielding similar results. **d** Analysis of ATR pT1989 phosphorylation in Tpr-depleted cells. Cells were transfected with siTPR #53 or control siRNA (siCTRL). Transfected cells were either non-treated (Mock) or treated with 2 mM hydroxyurea (HU) for 4 hours. ATR total was used as a loading control. Data are representative of two independent experiments. Multiple gels/blots were processed in parallel, ensuring equal and comparable loading across gels. Source data for **a, c,** and **d** are provided in the Source Data file.

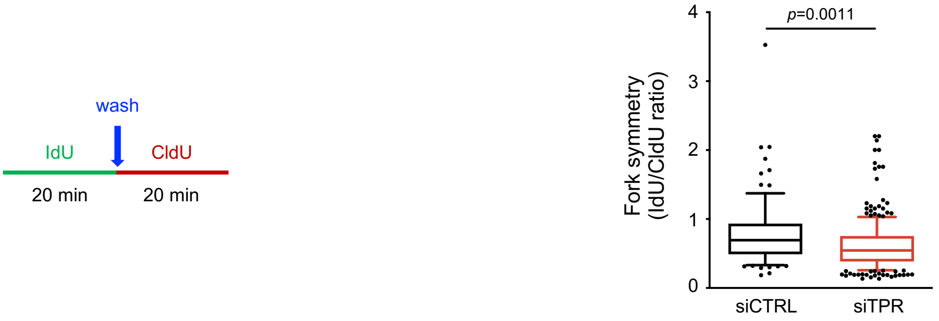

Supplementary Fig. 2 Tpr depletion impacts replication fork progression. DNA combing analysis of the replication fork symmetry in Tpr-depleted (siTPR) and control (siCTRL) HeLa cells in non-treated conditions. **Left:** Schematic of the IdU/CldU pulse-labeling protocol used. HeLa cells were pulse-labeled with IdU for 20 minutes, washed, and pulse-labeled with CldU for 20 minutes. IdU was detected using the specific primary antibody and the secondary antibody in green. CldU was detected using the specific primary antibody and the secondary antibody in red. **Right:** Analysis of the replication fork symmetry. IdU/CldU ratio obtained from siCTRL (n=89) and siTPR (n=267; red) fibers are plotted. The line within the box denotes the median and the box spans the interquartile range (25 to 75th percentiles). Whiskers extend from the 10 to 90th percentiles with outliers represented by circles. Statistical significance was tested using a two-tailed unpaired t-test with Welch's correction.

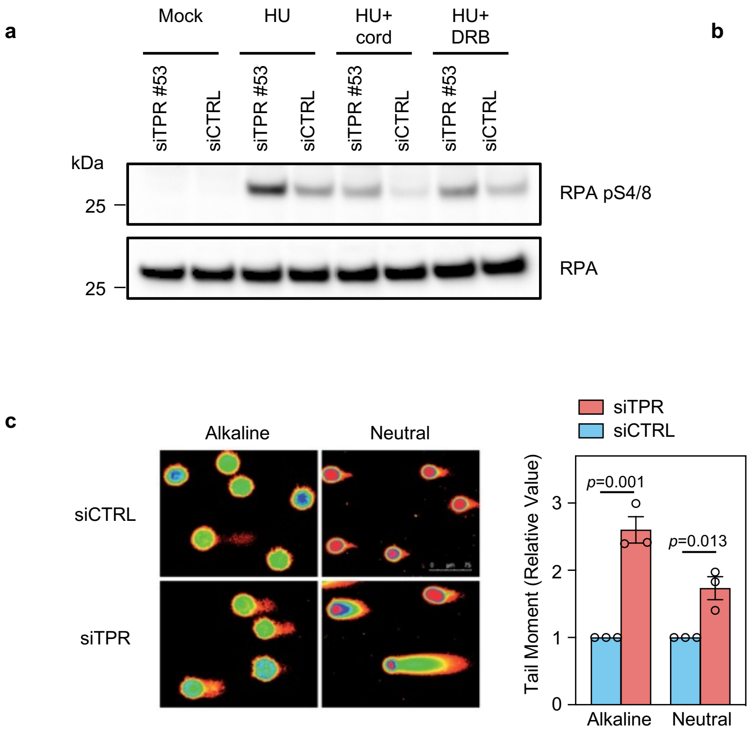

Supplementary Fig. 3 Tpr depletion leads to DNA-RNA hybrid accumulation. a DRB can rescue RPA pS4/8 phosphorylation upon hydroxyurea treatment in Tpr-depleted U-2 OS cells. Tpr-depleted (siTPR) and control (siCTRL) U-2 OS cells were either non-treated (Mock) or treated with 2 mM hydroxyurea (HU) for 4 hours. In parallel, cells were pre-treated with 50 µM cordycepin or 100 µM DRB for 3 hours, and then 2 mM HU was added for 4 hours. Staining of RPA total is used as a loading control. Data are representative of three independent experiments. Source data for **a** are provided in the Source Data file. **b** Representative images of γH2AX and 53BP1 foci detection by immunofluorescence in Tpr-depleted (siTPR) and control (siCTRL) HeLa cells from 3 independent experiments. γH2AX was detected in green and 53BP1 in red. Nuclei were stained with DAPI (blue). **c** DNA breaks measured by alkaline and neutral single-cell gel electrophoresis (comet assay) in Tpr-depleted (siTPR, red) and control (siCTRL, blue) HeLa cells. Representative images (*left*) and mean ± SEM of median comet tail moment (*right*) from independent experiments (n=3) are shown. Statistical analysis by two-tailed unpaired student t-test.

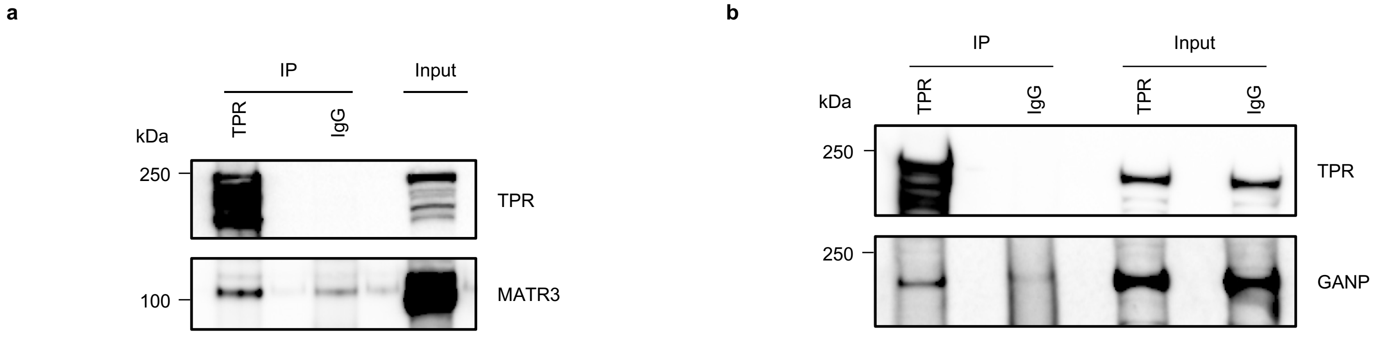

Supplementary Fig. 4 Tpr interacts with proteins involved in RNA metabolism. a Tpr interacts with MATR3. Endogenous Tpr protein was co-immunoprecipitated using Tpr antibody from U-2 OS cells. The experiment was performed twice yielding similar results. **b** Tpr interacts with GANP. Endogenous Tpr protein was co-immunoprecipitated using Tpr antibody from U-2 OS cells. The experiment was performed twice yielding similar results. Multiple gels/blots were processed in parallel, ensuring equal and comparable loading across gels. Source data for **a** and **b** are provided in the Source Data file.

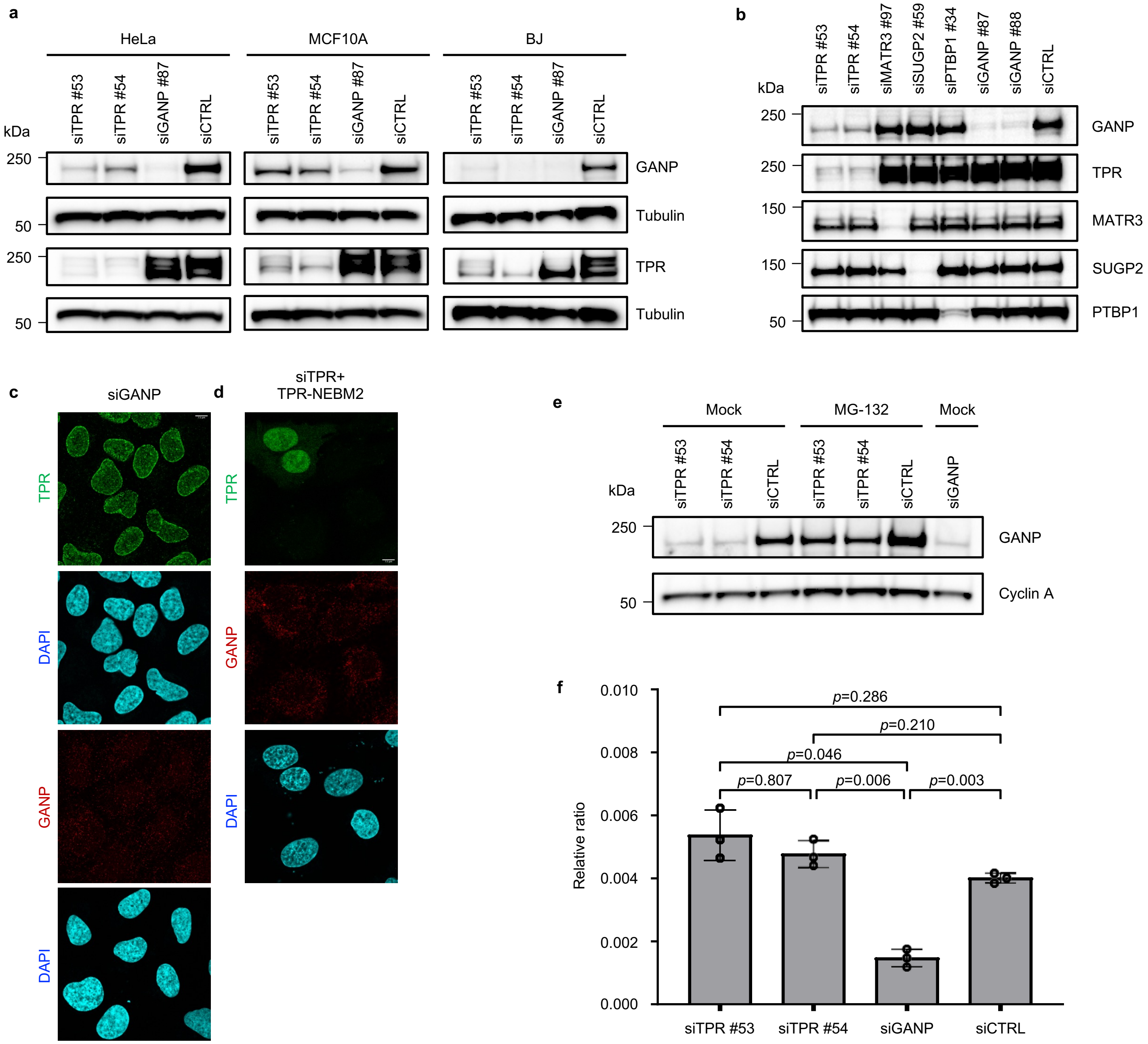

Supplementary Fig. 5 Tpr maintains normal protein levels of GANP. a Tpr depletion destabilizes GANP in BJ, HeLa, and MCF10A cells. Cells were transfected using siRNAs. Tpr and GANP protein levels were analyzed by immunoblotting. Tubulin serves as a loading control. The experiment was performed twice yielding similar results. **b** Depletion of MATR3, SUGP2, and PTBP1 do not impact GANP protein level. U-2 OS cells were transfected using siRNAs. Tubulin, loading control. The experiment was performed twice yielding similar results. **c** U-2 OS cells were transfected using siRNA targeting GANP (siGANP). Levels of Tpr and GANP were assessed using immunofluorescence. Nuclei were stained with DAPI. The scale bar represents 7.5 μm. Data are representative of three independent experiments. **d** Expression of siRNA-resistant full-length Tpr-NEBM (Nuclear Envelope Binding Mutant) is not able to rescue GANP localization at the nuclear envelope. U-2 OS cells were transfected using siRNA targeting endogenous Tpr (siTPR), and with siRNA-resistant Tpr-NEBM2-GFP (NEBM2) construct carrying L458D/ M489D double amino acid substitution in Tpr nuclear envelope binding domain. Levels of Tpr-NEBM2-GFP and GANP were assessed using immunofluorescence. Nuclei, DAPI. The scale bar represents 7.5 μm. Data are representative of three independent experiments. **e** Treatment of Tpr-depleted cells using proteasome inhibitor can restore GANP protein level. U-2 OS cells were transfected using siRNAs. 72 hours after siRNA transfection, cells were treated with 10 μM proteasome inhibitor (MG-132) for 4 hours. GANP protein levels were analyzed by immunoblotting. Accumulation of cyclin A is used as a positive control for MG-132 treatment. The experiment was performed twice yielding similar results. **f** Tpr depletion does not lead to a decrease in GANP mRNA level. U-2 OS cells were transfected using siRNAs, and GANP mRNA levels were analyzed using real-time PCR 72 hours after siRNA transfection. Relative quantification analysis was used to compare the target gene of interest (GANP) and the reference gene (GAPDH) and to express the final result as a ratio of these genes. Data are represented as mean ± SD from three independent experiments (n=3). Statistical analysis: Brown-Forsythe and Welch ANOVA with Dunnett's T3 post-analysis for multiple comparisons. Source data for **a, b, e** is provided in the Source Data file.

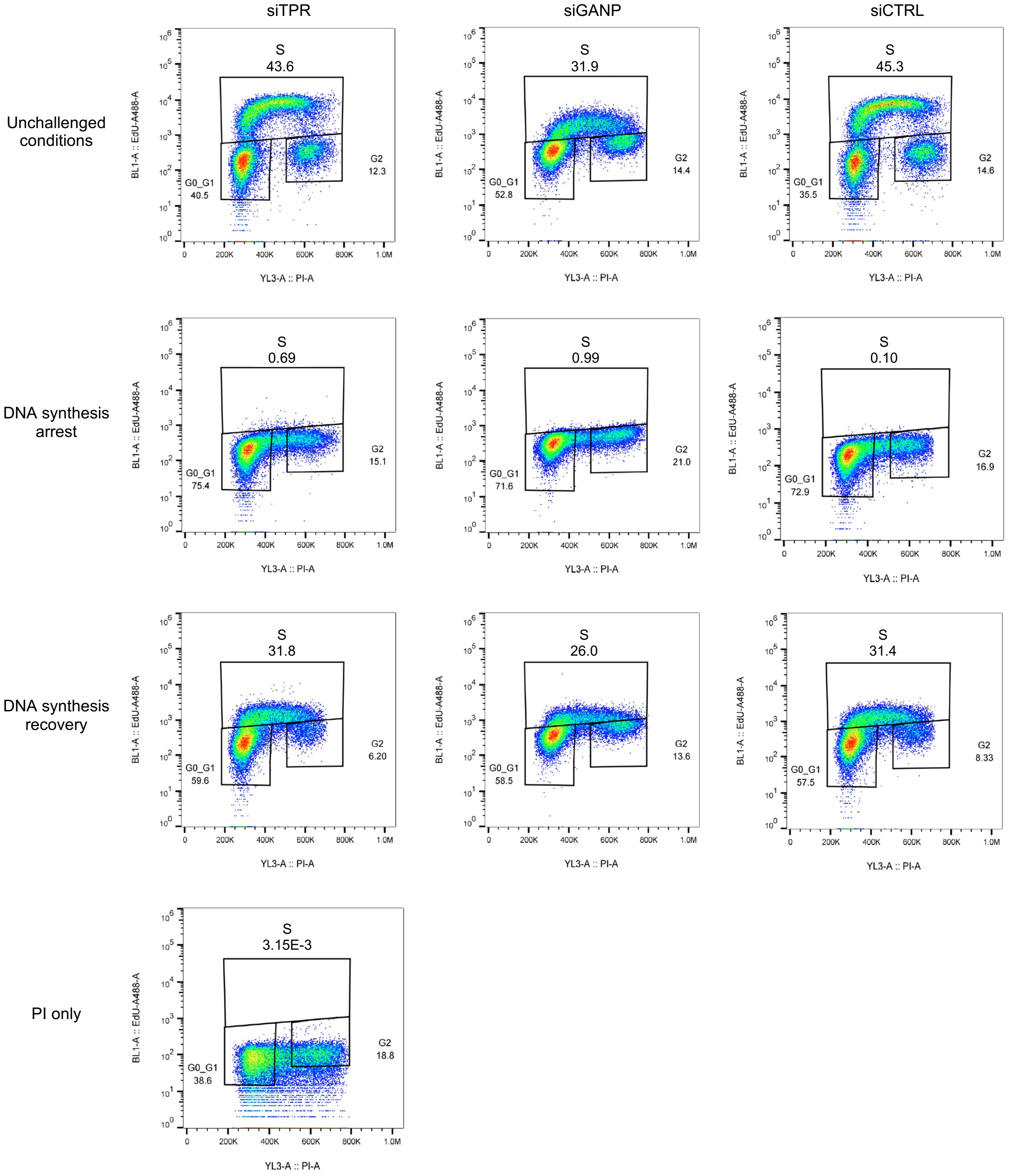

Supplementary Fig. 6 GANP-depleted cells exhibit replication defects. EdU profiles of TPR- and GANP-depleted U-2OS cells both in unchallenged conditions and in replication stress conditions that were used to measure replication fork speed and fork symmetry using DNA fibers analysis in Fig. 2a, b, c. In all experimental setups, cells were labeled with 10 μM EdU for 20 minutes. EdU was detected using Alexa Fluor 488, while total DNA content was stained using propidium iodide. Samples were analyzed by flow cytometry. Top: Experimental setup 1 (unchallenged conditions): TPR-depleted, GANP-depleted, Control cells labeled with EdU for 20 minutes. Middle: Experimental setup 2 (the ability of cells to arrest DNA synthesis in the presence of hydroxyurea): TPR-depleted, GANP-depleted, Control cells treated with 2 mM hydroxyurea for 4 hours. EdU was added for 20 minutes before the end of hydroxyurea treatment. Bottom: Experimental setup 3 (recovery of DNA synthesis upon hydroxyurea removal): TPR-depleted, GANP-depleted, Control cells treated with 2 mM hydroxyurea for 4 hours, washed and labeled with EdU for 20 minutes without hydroxyurea treatment. PI only (propidium iodide) is a figure exemplifying the gating strategy.

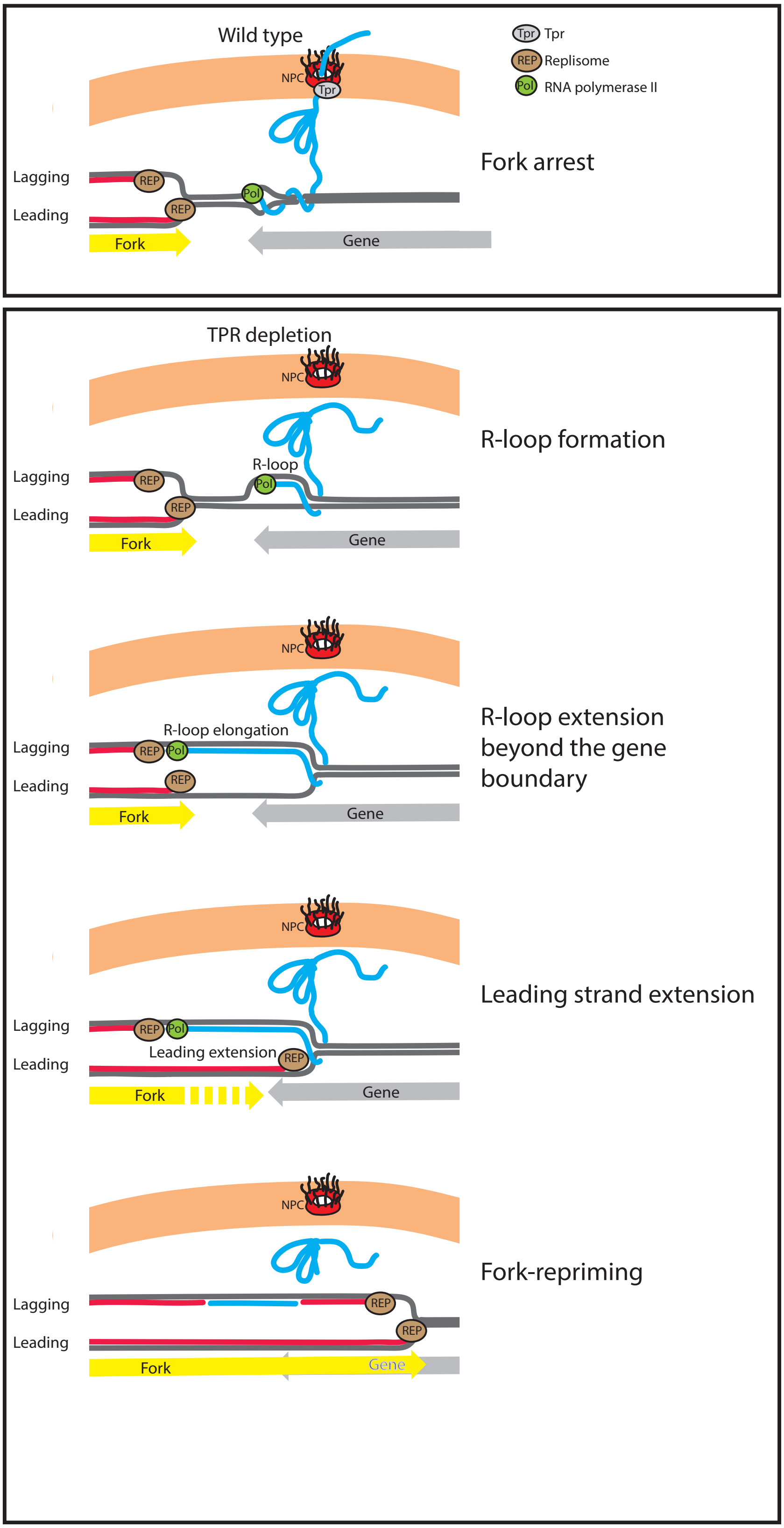

Supplementary Fig. 7 Model. A model for replication repriming in Tpr defective cells. In wild-type cells, the Tpr nucleoporin mediates the interaction of a network of proteins involved in splicing, RNA processing and export with nuclear pores, thus establishing physical connections between transcribed chromatin and the nuclear envelope. Following HU treatment, stalled replication forks arrest with the leading strand protruding. A replication fork encountering a gene unit head-on is shown. In Tpr depleted cells mRNA splicing and processing are uncoupled from export through the nuclear envelope, thus facilitating the formation at the end of the gene of DNA-RNA hybrids in the R-loop conformation. The R-loop can be further extended beyond the transcription termination site, transcribe the unreplicated DNA downstream of the fork and approach the 5' end of the lagging strand. The leading strand DNA polymerases then replicates the non-transcribed strand of the R-loop and, subsequently, new Okazaki fragments are synthesized downstream the DNA-RNA hybrid, thus re-establishing a normal fork. See text for further details.

Supplementary Tables

	SILAC I	SILAC II	SILAC III
Total number of proteins identified	1921	671	831
Number of significant proteins ($p<0.05$)	93	83	109

Supplementary Table 1 Total number of proteins and the number of significant proteins identified as Tpr-interactors in SILAC experiments. Significantly enriched Tpr-interacting proteins were calculated using the “Significance B” feature in Perseus, a function that calculates the extremes of the ratio distribution of the population, taking into account also the protein intensities and dividing the population in bins of 300 elements, to give a more accurate calculation.

siRNA		
siRNA	Source	Identifier
Silencer Select siRNA against ATR #536	Thermo Fisher Scientific	siRNA ID: s536
Silencer Select siRNA against TPR #53	Thermo Fisher Scientific	siRNA ID: s14353
Silencer Select siRNA against TPR #54	Thermo Fisher Scientific	siRNA ID: s14354
Silencer Select siRNA against TPR #55	Thermo Fisher Scientific	siRNA ID: 14355
Silencer Select siRNA against MATR #97	Thermo Fisher Scientific	siRNA ID: s18897
Silencer Select siRNA against MCM3AP #87	Thermo Fisher Scientific	siRNA ID: s16987
Silencer Select siRNA against MCM3AP #88	Thermo Fisher Scientific	siRNA ID: s16988
Silencer Select siRNA against MCM3AP #89	Thermo Fisher Scientific	siRNA ID: s16989
Silencer Select siRNA against PTBP1 #34	Thermo Fisher Scientific	siRNA ID: s11434
Silencer Select siRNA against SUGP2 #59	Thermo Fisher Scientific	siRNA ID: s19759
Silencer Select siRNA Negative Control #1	Thermo Fisher Scientific	Catalog number: 4390843
ON-TARGETplus Human TPR siRNA-SMARTpool	Horizon Discovery	Catalog ID: L-010548
ON-TARGETplus Non-targeting Pool	Horizon Discovery	Catalog ID: D-001810-10

Primers	
Sequence	Name
5’-GGCTCAGGTTGAGAGTCTCCGATACCGACAAAGGGTTGAACTT-3’	TPR_mut_si53_FWD
5’-AAGTTCAACCCTTTGTCGGTATCGGAGACTCTCAACCTGAGCC-3’	TPR_mut_si53_REV
5’- CCTTTCTGAAAAGGAAGTACACACAAAGCGTATTCAACAATTG-3’	TPR_mut_si54_FWD
5’- CAATTGTTGAATACGCTTTGTGTGTACTTCCTTTTCAGAAAGG-3’	TPR_mut_si54_REV

Supplementary Table 2 Oligonucleotides used in this study.
