# Supplementary material for: The human nucleoporin Tpr protects cells from RNA-mediated replication stress
Source: Nat Commun. 2021 Jun 24;12:3937. doi: 10.1038/s41467-021-24224-3 (PMC8225803; doi:10.1038/s41467-021-24224-3)
Supplement: Supplementary file 2 — Reporting Summary [file 41467_2021_24224_MOESM2_ESM.pdf]

nature research

Corresponding author(s): A. Aguilera, J. Bartek, M. Foiani

Last updated by author(s): May 12, 2021

Reporting Summary

Nature Research wishes to improve the reproducibility of the work that we publish. This form provides structure for consistency and transparency in reporting. For further information on Nature Research policies, see Authors & Referees and the Editorial Policy Checklist.

Statistics

For all statistical analyses, confirm that the following items are present in the figure legend, table legend, main text, or Methods section.

n/a	Confirmed	
☐	☒	The exact sample size (*n*) for each experimental group/condition, given as a discrete number and unit of measurement
☐	☒	A statement on whether measurements were taken from distinct samples or whether the same sample was measured repeatedly
☐	☒	The statistical test(s) used AND whether they are one- or two-sided<br>*Only common tests should be described solely by name; describe more complex techniques in the Methods section.*
☒	☐	A description of all covariates tested
☐	☒	A description of any assumptions or corrections, such as tests of normality and adjustment for multiple comparisons
☐	☒	A full description of the statistical parameters including central tendency (e.g. means) or other basic estimates (e.g. regression coefficient) AND variation (e.g. standard deviation) or associated estimates of uncertainty (e.g. confidence intervals)
☐	☒	For null hypothesis testing, the test statistic (e.g. *F*, *t*, *r*) with confidence intervals, effect sizes, degrees of freedom and *P* value noted<br>*Give P values as exact values whenever suitable.*
☒	☐	For Bayesian analysis, information on the choice of priors and Markov chain Monte Carlo settings
☒	☐	For hierarchical and complex designs, identification of the appropriate level for tests and full reporting of outcomes
☒	☐	Estimates of effect sizes (e.g. Cohen's *d*, Pearson's *r*), indicating how they were calculated

Our web collection on statistics for biologists contains articles on many of the points above.

Software and code

Policy information about availability of computer code

Data collection	Gatan Microscopy Suite Software, version 2.3.3 64 bit<br>Leica Application Suite X, version 3.5.2.18963<br>scanR software, version 2.3.0.7<br>Zeiss ZEN software, for LSM microscope image acquisition
Data analysis	CellProfiler, version 3.1.8<br>Enrich, http://amp.pharm.mssm.edu/Enrichr<br>Fiji, version 1.52u<br>GraphPad Prism, version 8.4.3.686<br>ImageJ, version 2.1.0/1.53C<br>LSM ZEN, version<br>LightCycler 96, version 1.1.0.1320<br>MaxQuant, version 1.5.2.8<br>Metamorph, version v7.5.1.0<br>Perseus, version 1.5.2.6<br>STRING, version 11.0<br>ZEISS ZEN lite 2012 software, analysis of LSM-acquired micrographs

For manuscripts utilizing custom algorithms or software that are central to the research but not yet described in published literature, software must be made available to editors/reviewers. We strongly encourage code deposition in a community repository (e.g. GitHub). See the Nature Research guidelines for submitting code & software for further information.

Data

Policy information about availability of data

All manuscripts must include a data availability statement. This statement should provide the following information, where applicable:

- Accession codes, unique identifiers, or web links for publicly available datasets
- A list of figures that have associated raw data
- A description of any restrictions on data availability

The datasets underlying proteomic analysis generated and analyzed during the current study are available in the Peptide Atlas repository, http://www.peptideatlas.org. Dataset Identifier: PASS01419. [https://db.systemsbiology.net/sbeams/cgi/PeptideAtlas/PASS_View?identifier=PASS01419]
Source data underlying Figures are provided in the source data file.

Field-specific reporting

Please select the one below that is the best fit for your research. If you are not sure, read the appropriate sections before making your selection.

☒ Life sciences ☐ Behavioural & social sciences ☐ Ecological, evolutionary & environmental sciences

For a reference copy of the document with all sections, see nature.com/documents/nr-reporting-summary-flat.pdf

Life sciences study design

All studies must disclose on these points even when the disclosure is negative.

Sample size	Sample size for the experiments shown was chosen to obtain statistical power, in conformity to accepted standard sample size in a number of previous publications using these approaches:<br>Lukas C, et al. 53BP1 nuclear bodies form around DNA lesions generated by mitotic transmission of chromosomes under replication stress. Nature cell biology 13, 243-253 (2011).<br>Giannattasio M, Zwicky K, Follonier C, Foiani M, Lopes M, Branzei D. Visualization of recombination-mediated damage bypass by template switching. Nature structural & molecular biology 21, 884-892 (2014).<br><br>For DNA fiber experiments, if equivalent experiments were not different statistically (p-value of t-test <0.001), the total number of DNA fibers from both experiments is presented.<br><br>Untreated control cells, with drug- vehicle, or cells transfected with Non-Targeting (NT kd) siRNA control were included as appropriate controls for each specific experiment.
Data exclusions	No exclusion was applied.
Replication	For all experiments, the number of biological replicates is indicated and reproduced the representative data are shown in figures.
Randomization	We were working with asynchronously growing cell populations or individual DNA replication molecules from these cell populations, therefore further randomization was not necessary for our approaches.
Blinding	Individual repetitions for the Clonogenic cell survival assay, DNA fiber analysis and Electron microscopy analysis were blinded to the investigators. For the automated acquisition of 53BP1 foci blinding was not necessary due to intrinsically unbiased nature of the approach.

Reporting for specific materials, systems and methods

We require information from authors about some types of materials, experimental systems and methods used in many studies. Here, indicate whether each material, system or method listed is relevant to your study. If you are not sure if a list item applies to your research, read the appropriate section before selecting a response.

Materials & experimental systems

n/a	Involved in the study	
☐	☒	Antibodies
☐	☒	Eukaryotic cell lines
☒	☐	Palaeontology
☒	☐	Animals and other organisms
☐	☒	Human research participants
☒	☐	Clinical data

Methods

n/a	Involved in the study	
☒	☐	ChIP-seq
☐	☒	Flow cytometry
☒	☐	MRI-based neuroimaging

Antibodies

Antibodies used

DNA fiber analysis:
BrdU (Bio-Rad, #OBT0030, 1:1000)
BrdU (BD Biosciences, #347580, 1:1000)
Alexa Fluor 568 (Thermo Fisher Scientific, # A-11077, 1:500)
Alexa Fluor 488 (Thermo Fisher Scientific, #A-21202, 1:500)

Immunoblotting:
ATR (Cell Signaling, #2790, 1:1000)
phospho-ATR (Thr1989) (Cell Signaling, #58014, 1:1000)
Chk1 (Santa Cruz Biotechnology, #sc-8408, 1:1000)
phospho-Chk1 (Ser345) (Cell Signaling, #2348, 1:1000)
phospho-Chk1 (Ser317) (Cell Signaling, #12302, 1:1000)
Cyclin A2 (Abcam, #ab16726, 1:000)
GANP (Abcam, #ab113295, 1:1000)
GANP (Sigma-Aldrich, #HPA021527, 1:1000)
GFP (Amsbio, TP401, 1:2500)
MATR3 (Bethyl, #A300-591A, 1:1000)
PTBP1 (Thermo Fisher Scientific, #32-4800, 1:000)
RPA32 (Abcam, #ab16855, 1:2000)
phospho-RPA32 (S4/S8) (Bethyl, #A300-245A, 1:1000)
SUGP2 (Sigma-Aldrich, #HPA061111, 1:1000)
Tpr (Sigma-Aldrich, #HPA019661, 1:1000)
tubulin (Sigma-Aldrich, #T5168, 1:5000)
Goat Anti-Mouse IgG (H + L)-HRP Conjugate (BIO-RAD, #1706516, 1:5000)
Goat Anti-Rabbit IgG (H + L)-HRP Conjugate (BIO-RAD, #1706515, 1:5000)

Immunostaining:
Cyclin A2 (Abcam, #ab16726, 1:200)
Flag M2 (Sigma-Aldrich, #F1804, 1:100)
GANP (Sigma-Aldrich, #HPA021527, 1:100)
phospho-H2A.X (Ser139) (Millipore JBW301, #05-636, 1:500)
Nucleolin (Abcam, #ab50279, 1:1000)
S9.6 (Hybridoma cell line HB-8730, 1:100)
Tpr (Sigma-Aldrich, #HPA019661, 1:100)
53BP1 (Novus Biologicals, #NB100-304, 1:500)
Alexa Fluor 488 (Thermo Fisher Scientific, #A-21206, 1:400),
Alexa Fluor 488 (Molecular Probes, #A11008 and #A11029, 1:1000),
Alexa Fluor 546 (Molecular Probes, #A21123, 1:1000),
Alexa Fluor 568 (Molecular Probes, #A11011, 1:1000),
Alexa Fluor 594 (Molecular Probes, #A21201, 1:1000),
Alexa Fluor 647 (Molecular Probes, #A21244 and #A21463, 1:1000)
Cy3 (Jackson ImmunoResearch Laboratories, #715-165-150, 1:400)

Immunohistochemical analysis:
Tpr (Sigma-Aldrich, #HPA019661, 1:1000)
Tpr (Sigma-Aldrich, #HPA024336, diluted 1:250)
GANP (Sigma-Aldrich, #HPA021527, diluted 1: 250)

Validation

All primary antibodies used in this study are commercially available and show the band of the expected size. In addition, the following antibodies were further validated for our approaches using siRNA directed against the protein of interest in U-2OS cells:
ATR (Cell Signaling, #2790), siRNA (Thermo Fisher Scientific, siRNA ID: s536)
phospho-ATR (Thr1989) (Cell Signaling, #58014), siRNA (Thermo Fisher Scientific, siRNA ID: s536)
Tpr (Sigma-Aldrich, #HPA019661), siRNAs (Thermo Fisher Scientific, siRNAs ID: s14353, s14354, 14355)
GANP (Sigma-Aldrich, #HPA021527), siRNAs (Thermo Fisher Scientific, siRNAs ID: s16987, s16988, s16989)
MATR3 (Bethyl, #A300-591A), siRNA (Thermo Fisher Scientific, siRNA ID: s18897)
PTBP1 (Thermo Fisher Scientific, #32-4800), siRNA (Thermo Fisher Scientific, siRNA ID: s11434)
SUGP2 (Sigma-Aldrich, #HPA061111), (Thermo Fisher Scientific, siRNA ID: s19759)

Following primary antibodies used in this study were also validated by the companies and/or cited in the research papers:
DNA fiber analysis:
BrdU (Bio-Rad, #OBT0030, 1:1000), cited in 238 research papers
BrdU (BD Biosciences, #347580, 1:1000), cited in 736 research papers

Immunoblotting:
ATR (Cell Signaling, #2790, 1:1000), cited in 84 research papers
phospho-ATR (Thr1989) (Cell Signaling, #58014, 1:1000), cited in 5 research papers
Chk1 (Santa Cruz Biotechnology, #sc-8408, 1:1000), cited in 621 research papers
phospho-Chk1 (Ser345) (Cell Signaling, #2348, 1:1000), cited in 487 research papers
phospho-Chk1 (Ser317) (Cell Signaling, #12302, 1:1000), cited in 63 research papers
Cyclin A2 (Abcam, #ab16726, 1:000), cited in 27 research papers

GANP (Abcam, #ab113295, 1:1000), cited in 1 research paper
GANP (Sigma-Aldrich, #HPA021527, 1:1000), validated by the company and cited in 1 research paper
GFP (Amsbio, TP401, 1:2500), cited in 21 research papers
MATR3 (Bethyl, #A300-591A, 1:1000), cited in 25 research papers
PTBP1 (Thermo Fisher Scientific, #32-4800, 1:000), cited in 36 research papers
RPA32 (Abcam, #ab16855, 1:2000), cited in 10 research papers
phospho-RPA32 (S4/S8) (Bethyl, #A300-245A, 1:1000), cited in 250 research papers
SUGP2 (Sigma-Aldrich, #HPA061111, 1:1000), validated by the company
Tpr (Sigma-Aldrich, #HPA019661, 1:1000), validated by the company and cited in 3 research papers
tubulin (Sigma-Aldrich, #T5168, 1:5000), cited in 3048 research papers

Immunostaining:
Cyclin A2 (Abcam, #ab16726, 1:200), cited in 27 research papers
Flag M2 (Sigma-Aldrich, #F1804, 1:100), cited in 5110 research papers
GANP (Sigma-Aldrich, #HPA021527, 1:100), validated by the company and cited in 1 research paper
phospho-H2A.X (Ser139) (Millipore JBW301, #05-636), validated by the company and cited in 2303 research papers
Nucleolin (Abcam, #ab50279, 1:1000), validated by the company and cited in 18 research papers
S9.6 (Hybridoma cell line HB-8730, 1:100), cited in 5 research papers
Tpr (Sigma-Aldrich, #HPA019661, 1:100), validated by the company and cited in 3 research papers
53BP1 (Novus Biologicals, #NB100-304, 1:500), validated by the company and cited in 427 research papers

Immunohistochemical analysis:
Tpr (Sigma-Aldrich, #HPA019661, 1:1000), validated by the company and cited in 3 research papers
Tpr (Sigma-Aldrich, #HPA024336, diluted 1:250), validated by the company and cited in 3 research papers
GANP (Sigma-Aldrich, #HPA021527, diluted 1: 250), validated by the company and cited in 1 research paper

Eukaryotic cell lines

Policy information about cell lines

Cell line source(s)

HeLa cells (European Collection of Authenticated Cell Cultures: ECACC)
U-2 OS cells (ATCC)
BJ cells (ATCC)
MCF10A cells (ATCC)

Authentication

Cell lines were purchased from ECACC or ATCC and authenticated by STR method.

Mycoplasma contamination

All cell lines used in this study were negative for mycoplasma. We regularly check by PCR the presence of mycoplasma in all cell lines used in our laboratory.

Commonly misidentified lines
(See ICLAC register)

No commonly misidentified cell lines were used in this study.

Human research participants

Policy information about studies involving human research participants

Population characteristics

The patient population consisted of Caucasian females from Spain, aged 25 to 86 years (mean 54.8 years). The patients were not treated before the surgical operations, which were performed in the period 1995-2005. No genetic information was available on either the patients or the ovarian tumors.

Recruitment

These were consecutive cases of tumors surgically resected during the period 1995-2005, and the archival paraffin blocks from the tumors stored at the Department of Pathology, University Hospital, Las Palmas, Gran Canaria. The only selection that was undertaken was to examine only invasive serous carcinoma types in this retrospective immunohistochemical study, in order to ensure a homogenous cohort of the most common type of ovarian malignancy (i.e. other types of ovarian tumors from the above period were not included). All patients provided written informed consent for the usage of their tissue specimens for research at the time of surgical operation. There was no self-selection or other bias in selecting the archival paraffin specimens for this study, except for the histologically diagnosed serous carcinoma type.

Ethics oversight

The study was performed in accordance with the Spanish codes of conduct (Ley de Investigacio ́ n Biomedica) and with the World Medical Association Declaration of Helsinki, after approval by the review board of the participating institution: the University Hospital, Las Palmas, Gran Canaria, Spain.

Note that full information on the approval of the study protocol must also be provided in the manuscript.

Flow Cytometry

Plots

Confirm that:

☒ The axis labels state the marker and fluorochrome used (e.g. CD4-FITC).

☒ The axis scales are clearly visible. Include numbers along axes only for bottom left plot of group (a 'group' is an analysis of identical markers).

☒ All plots are contour plots with outliers or pseudocolor plots.

☒ A numerical value for number of cells or percentage (with statistics) is provided.

Methodology

Sample preparation	U-2 OS cells were labeled with 10 µM EdU for 20 minutes. Next, cells were fixed using 3.6% PFA in 1 x PBS for 15 min and permeabilized with 0.25% Triton-X for at room temperature for 15 min. S-phase cells were detected using Click-iT EdU Alexa Fluor 488 Imaging Kit (Invitrogen, #C10337) according to the manufacturer's protocol, and total DNA content was stained using propidium iodide (2.5 µg/ml) in the presence of RNase A (250 µg/ml). Samples were analyzed by flow cytometry.
Instrument	FACS ATTUNE NXT THERMO FISHER
Software	Attune Nxt for acquisition and FLOWJO 10 for analysis
Cell population abundance	*Describe the abundance of the relevant cell populations within post-sort fractions, providing details on the purity of the samples and how it was determined.*
Gating strategy	PI-positive cells (gated cell cycle without doublets) were analyzed for Edu-A488 positive and negative

☒ Tick this box to confirm that a figure exemplifying the gating strategy is provided in the Supplementary Information.
